# Cantharidin inhibits osteosarcoma proliferation and metastasis by directly targeting miR-214-3p/DKK3 axis to inactivate β-catenin nuclear translocation and LEF1 translation

**DOI:** 10.7150/ijbs.51638

**Published:** 2021-06-16

**Authors:** Shaopu Hu, Junli Chang, Hongfeng Ruan, Wenlan Zhi, Xiaobo Wang, Fulai Zhao, Xiaoping Ma, Xingyuan Sun, Qianqian Liang, Hao Xu, Yongjun Wang, Yanping Yang

**Affiliations:** 1 Longhua Hospital, Shanghai University of Traditional Chinese Medicine, Shanghai, 200032, China.; 2 Key laboratory of theory and therapy of muscles and bones, Ministry of Education, Shanghai, 200032, China.

**Keywords:** Cantharidin, osteosarcoma, Wnt/β-catenin, miR-214-3p, DKK3

## Abstract

**Background:** As the leading primary bone cancer in adolescents and children, osteosarcoma patients with metastasis show a five-year-survival-rate of 20-30%, without improvement over the past 30 years. Wnt/β-catenin is important in promoting osteosarcoma development. DKK3 is a Wnt/β-catenin antagonist and predicted to have the specific binding site in 3′-UTR with miR-214-3p.

**Methods:** miR-214-3p and DKK3 levels were investigated in human osteosarcoma tissues and cells by RT-qPCR; the prognostic importance of DKK3 level in osteosarcoma patients was determined with Log-rank test; direct binding between DKK3 with miR-214-3p was identified with targetscan; anti-osteosarcoma mechanism of cantharidin was investigated by miR-214-3p silence/over-expression with or without cantharidin treatment, and nuclear/cytoplasmic protein assay in osteosarcoma cells.

**Results:** Down-regulated DKK3 indicated poor prognosis of osteosarcoma patients. Up-regulated miR-214-3p promoted proliferation and migration, while suppressed apoptosis of osteosarcoma cells by increasing β-catenin nuclear translocation and LEF1 translation via degradation of DKK3. Cantharidin suppressed viabilities, migration and invasion, while promoted cell cycle arrest and apoptosis in 143B and U-2 OS cells via down-regulating miR-214-3p to up-regulate DKK3, thus inhibited p-GSK-3β expression, β-catenin nuclear translocation and LEF1 translation. Meanwhile, cantharidin inhibited tumor growth in xenograft-bearing mice with 143B cell injection in tibia.

**Conclusion:** miR-214-3p mediated Wnt/β-catenin/LEF1 signaling activation by targeting DKK3 to promote oncogenesis of osteosarcoma; cantharidin inhibited proliferation and metastasis of osteosarcoma cells via down-regulating miR-214-3p to up-regulate DKK3 and decrease β-catenin nuclear translocation, indicating that cantharidin may be a prospective candidate for osteosarcoma treatment by targeting miR-214-3p/DKK3/β-catenin signaling.

## Introduction

Osteosarcoma is the leading primary bone cancer, which is diagnosed mainly in adolescents and children with an extremely destructive phenotype. Osteosarcoma is frequently originates from the long bones, including distal femur (30%), proximal humerus (15%), and proximal tibia (15%) 1-6.

Current standard therapeutic strategies, the combination of preoperative neoadjuvant chemotherapy with limb salvage surgical procedure and postoperative chemotherapy, has improved outcome of osteosarcoma patients with non-metastatic disease, achieving a five-year-survival-rate of about 70% 7-10. However, the five-year-survival-rate of those osteosarcoma cases with metastatic disease is only 20% to 30% 11-13. Moreover, about 20% of the osteosarcoma patients already have pulmonary metastases, and 90% of them have a micrometastatic lesion at the time of diagnosis 14, 15, and 80% of the osteosarcoma patients will eventually have metastases even after the surgical treatment 16. Meanwhile, the clinical outcome and therapeutic strategies for osteosarcoma patients have changed very little over the past 30 years, the existing severe side effects and complications further limit the efficacy of these managements. Consequently, to develop novel treatments for improving osteosarcoma patient prognosis is crucial 2.

Wnt/β-catenin signaling is an evolutionary, highly conserved pathway which is important in cell differentiation, migration and proliferation 17-19. Activated Wnt/β-catenin signaling stimulates oncogenesis and development of various human malignancies, including osteosarcoma 19, 20. As the key factor of canonical Wnt signaling, β-catenin is accumulated in cytoplasm after binding to the Frizzled family receptor and experiences nuclear translocation. In the nuclear, β-catenin promotes the expression of Wnt-target genes by serving as the transcriptional coactivator of TCF/LEF. Cytoplasmic β-catenin experiences ubiquitination and proteasomal degradation when the Wnt signaling is inhibited 17-19. As a member of DKK family encoding secreted proteins, DKK (Dickkopf Homolog) 3 is a Wnt ligand activity antagonist and acts as an extracellular Wnt/β-catenin inhibitor 21, 22.

MicroRNAs (miRNAs) and their function in regulating the pathogenesis of malignant tumors have been a revolutionary discovery over the last decade in the molecular oncology field. The miRNA dysregulation is now considered to impact the key signaling pathways involved in cancer development, angiogenesis and metastasis in many cancers; therefore, regulating miRNA levels in cancer cells has promising potential as a therapeutic strategy 23, 24. Since microRNAs play crucial roles in down-regulating gene expression by binding to their mRNA targets 25, we predicted the potential miRNA having the complementary sequence with the 3'- untranslated region (UTR) of DKK3 by bioinformatics analysis using the online tool targetscan. As a result, miR-214-3p was identified to have potential binding sites with DKK3 3′-UTR, suggesting that miR-214-3p may directly interact with DKK3. Therefore, we hypothesized that up-regulating DKK3 via down-regulating miR-214-3p will achieve an inhibitory outcome on Wnt/β-catenin signaling activity, which will be a promising strategy to develop novel treatments for osteosarcoma patients. However, whether the miR-214-3p/DKK3/Wnt/β-catenin axis involves in osteosarcoma progression has not been reported.

Cantharidin (molecular formula for C_10_H_12_O_4_; chemical structure in Supplementary Fig. 1; nuclear magnetic resonance spectrum in supplementary Fig. 2) is a sesquiterpenoid bioactive substance extracted from the blister beetle of the genus *Mylabris*, which has been applied for more than 2000 years in traditional Chinese medicine 26, 27. Studies have reported the cytotoxic effects of cantharidin on several cancers, such as hepatocellular 28, 29, gastric 30, 31, breast 32, 33 and colorectal cancers 34, 35. However, the miR-214-3p/DKK3/GSK-3β/β-catenin/LEF1 axis related pharmacological mechanism of cantharidin on osteosarcoma remains unknown.

The aim of our current work was to explore oncogenic function of miR-214-3p/DKK3/Wnt/β-catenin axis in osteosarcoma. Then the anti-osteosarcoma effects of cantharidin both *in vivo* and *in vitro,* as well as the specific contribution of miR-214-3p/DKK3/GSK-3β/β-catenin/LEF1 axis were further investigated.

## Materials and Methods

### Reagents

RIPA Cell Lysis Buffer (Beyotime Institute of Biotechnology, Shanghai, China). SuperSignal Chemiluminescent HRP Substrate (Thermo Fisher scientific Inc, Rockford, US). PrimeScript RT reagent kit and SYBR Premix Ex Taq II (TaKaRa, Dalian, China). Matrigel, FITC Annexin V and propidium iodide (BD Biosciences, Bedford, US). Doxorubicin (DOX) (Sangon Biotech, Shanghai, China) with ultra-purified H_2_O as solvent. Cantharidin (Institute for Drug Control, Shanghai, China) with dimethyl sulfoxide (DMSO) as solvent. Trypsin-EDTA, Lipofectamine™ RNAiMAX and TRIzol reagent (Invitrogen, Rockville, US). CHIR99021 and antibody against β-actin (Sigma-Aldrich, Saint Louis, US). Fetal bovine serum (FBS. Gibco, Grand Island, US). U6 snRNA Real-time RT-PCR Normalization kit (GenePharma, Shanghai, China). Cell cycle detection kit (Key GENBioTECH, Nanjing, China). Antibodies against Bcl-2, cleaved PARP, Cyclin E2, CyclinB1, Myt1, Phospho-cdc2 (Tyr15), Phospho-Histone H3 (Ser10), Phospho-Wee1 (Ser642), Vimentin, DKK3, LEF1, GSK-3β, active β-catenin, total β-catenin and phospho-GSK-3β(Cell Signaling Technology, Danvers, US). Nuclear and Cytoplasmic Extraction Kit (Kangwei Century Company, Beijing, China). Transwell (Corning Incorporated, New York, US). Bicin choninic acid (BCA) Protein Assay Kit (abcam, Cambridge, US).

### Cell incubation

Human osteosarcoma (U-2 OS, 143B, Saos-2, MG-63 and MNNG) and osteoblastic (hFOB1.19) cells were provided by American Type Culture Collection (Manassas, US) and Cell Bank of Chinese Academy of Sciences (Shanghai, China), respectively. All cells were incubated with growth medium comprising FBS (10%), streptomycin (100 μg/ml) and penicillin (100 U/ml) in a 5% CO_2_-humidified incubator. Osteosarcoma cells were cultured at 37 °C with Mcosy'5a for Saos-2; RPMI 1640 for U-2 OS; modified Eagle medium (MEM) for 143B, MG-63 and MNNG. The hFOB1.19 cells were cultured at 34 °C with DMEM/F-12.

### Public data collection

Data about mRNA microarray and survival were obtained from Therapeutically Applicable Research to Generate Effective Treatments (TARGET, https://ocg.cancer.gov/programs/target).

### Cell transfection

Negative control (NC), miR-214-3p inhibitor (AntagomiR-214-3p) and miR-214-3p mimic (AgomiR-214-3p) were provided by GenePharma (Shanghai, China). Lipofectamine™ RNAiMAX was used for cell transfections.

### Cell viability test

Viability of cells (2×10^3^/100 μl/well) plated in specific xCELLigence-E-plate was continuously monitored in real time using an xCELLigence-Real-Time Cell Analyzer (Roche Applied Science, Mannheim, Germany).

### Apoptosis investigation by flow cytometer

Apoptotic rate was explored using Annexin V-FITC and PI Apoptosis Detection Kit. Briefly, cells (2×10^5^/well/2 ml) in 6-well plate were exposed to cantharidin at different concentration of 1.25, 2.5 or 5 μM for 24 h, with DMSO (1/10,000 V/V) as solvent control. After being cultured at room temperature for 30 min in dark with FITC-labeled Annexin-V, cells were then cultured with propidium iodide on ice for 5 min. Percentage of the Annexin V-FITC stained apoptotic cells were calculated after the flow cytometric analysis (Accuri C6, BD Biosciences, US).

### Cell cycle determination by flow cytometer

Cell Cycle Detection kit was used for cell cycle distribution assay. Briefly, cells exposed to cantharidin at different concentrations (0, 1.25, 2.5 or 5 μM) for 24 h were fixed overnight in 70% cold ethanol and incubated at 37 °C for 30 min with RNaseA, followed by culture with propidium iodide for 30 min at 4 °C in dark. Cell cycle distribution at G0/G1, S, and G2/M phases was detected with a flow cytometer.

### Real time migration and invasion assay

xCELLigence RTCA DP system was applied to evaluate invasion and migration of osteosarcoma cells. Briefly, specific CIM-Plate in xCELLigence RTCA DP system with 10% FBS containing culture medium in the lower chamber (165 μl/well) and different concentrations of cantharidin (40 μl/well) in the upper chamber was used to determine background. Then cells in serum-free medium (2×10^4^/100 μl/well) were plated in upper chamber. Cell migration ability was monitored continuously for 24 h. For cell invasion ability assay, the same condition as migration assay was used except for applying matrigel (1:40 dilution) pre-coated upper chambers and continuously monitoring for 48 h.

### Transwell migration examination

Cells plated in upper chamber of 12-well chamber plates (5×10^4^/well/200 μl serum-free culture medium) with chemotactic culture medium (10% FBS, 300 μl) in lower chamber were cultured for 24 h. Cells migrated to opposite side of chamber were washed two times softly with PBS, fixed for 30 min in 4% paraformaldehyde, stained for 10 min in 1% crystal violet solution, and photographed under microscope.

### Colony formation evaluation

Cells (1000/well) in 6-well plate were cultured overnight before being exposed to different treatment conditions for 24 h, and cultured in complete medium for additional seven days. Colonies were fixed for 10 min at room temperature in 10% formalin and stained for 15 min in crystal violet.

### Western blot investigation

RIPA Cell Lysis Buffer was used to lyse osteosarcoma cells on ice for total protein extraction according to manufacturer's guidelines. After centrifuging for 15 min at 4 °C and 13,000 g, supernatant (total protein) was harvested.

The NC-nuclear/plasma protein extraction kit was applied to isolate nuclear and plasma proteins from osteosarcoma cells following instructions. A 200 μl of pre-cooled reagent A (containing 1% protease inhibitor) and 11 μl reagent B were added into each 1.5ml EP tube containing collected cells, shaken and mixed well, then centrifuged at 12000 r/min and 4 °C for 15 min after keeping on ice for 1 minute, supernatant (protein in cytoplasm) was transferred into a fresh EP tube; the precipitation was then re-suspended in 100 μl reagent C (containing 1% protease inhibitor) on ice to crack for 40 minutes with shaking and mixing every 10 minutes, and the supernatant (nuclear proteins) was collected after centrifugation at 12000 r/min and for 15 min, 4 °C.

BCA method was applied for protein concentration determination. Proteins (20 μg/sample) were loaded on SDS-PAGE for separation, followed by transferring to PVDF membrane, blotting with primary and secondary antibodies respectively, visualizing with SuperSignal Chemiluminescent HRP Substrate and photographing with software Image Lab version in ChemiDoc MP Imaging System (Bio-Rad, US).

### RT-qPCR assay

Trizol reagent was applied for total cellular RNA purification. miR-214-3p expression was measured with U6 snRNA Real-time RT-PCR Normalization kit following the protocol. PrimeScript RT reagent kit was applied for Reverse transcription PCR according to the protocol. RT-qPCR was achieved on Bio-Rad CFX 96 real-time PCR system (Richmond, US) with SYBR Premix Ex Taq II. Traditional 2^-ΔΔCt^ method was applied to estimate relative RNA expression. *U6* and *gapdh* served as internal references for miR-214-3p and mRNAs, respectively.

### Xenograft tumorigenesis and treatment

Shanghai SLAC Laboratory Animal Co, Ltd. (Shanghai, China) provided 4-week-old female BALB/c nude mice were housed in separated cages and pathogen-free environment at Animal Experiment Centre of Shanghai University of Traditional Chinese Medicine.

A total of 10 μl logarithmic phase 143B cells mixed with matrigel (2×10^7^ cells/ml) were injected to left proximal tibia of each nude mouse after being anesthetized. Mice were separated into 3 groups randomly after 24 h for designated treatment, a control group, a cantharidin group and a DOX group (n=9).

Mice in the cantharidin group received intraperitoneal injection of 100 μl cantharidin (2.5 mg/kg·weight), DOX group received intraperitoneal injection of 100 μl doxorubicin (DOX) (1 mg/kg**·**body weight) and control group received intraperitoneal injection of same volume of DMSO in saline (1/10000V/V), one time/day. Tumor growth and mouse survival were monitored daily; the long diameters (a) and the short diameters (b) of the tumor masses were determined every seven days with a caliper for tumor volume (V) calculation: V =1/2×a×b^2^. Body weights of mice were determined every three days. Orthotopic tumor growth was dynamically monitored once a week with X-ray using the Image Station In-Vivo FX system (Kodak, Japan). After a continuous 28-day treatment, mice were sacrificed under the euthanasia by mask inhalation of 1.5% vaporized isoflurane to isolate and weigh the tumor masses.

Each experimental procedure was carried out following the regulation of Shanghai University of Traditional Chinese Medicine on using animals for laboratory experiments by the Animal Care and Use Committee.

### Statistics analysis

Measurement data in current work are represented as mean ± standard deviation (SD). GraphPad Prism 8.0 was used for statistics analyses. Differences between two groups were evaluated by *t-*test; one-way ANOVA was used for multi-group comparison; and the two survival curves were compared using the more standard Log-rank test (Mantel-Cox). *p< 0.05, **p< 0.01 and ***p < 0.001 specify statistical significance.

## Results

### miR-214-3p is increased in human osteosarcoma cells and tissues

**Figure 1:**
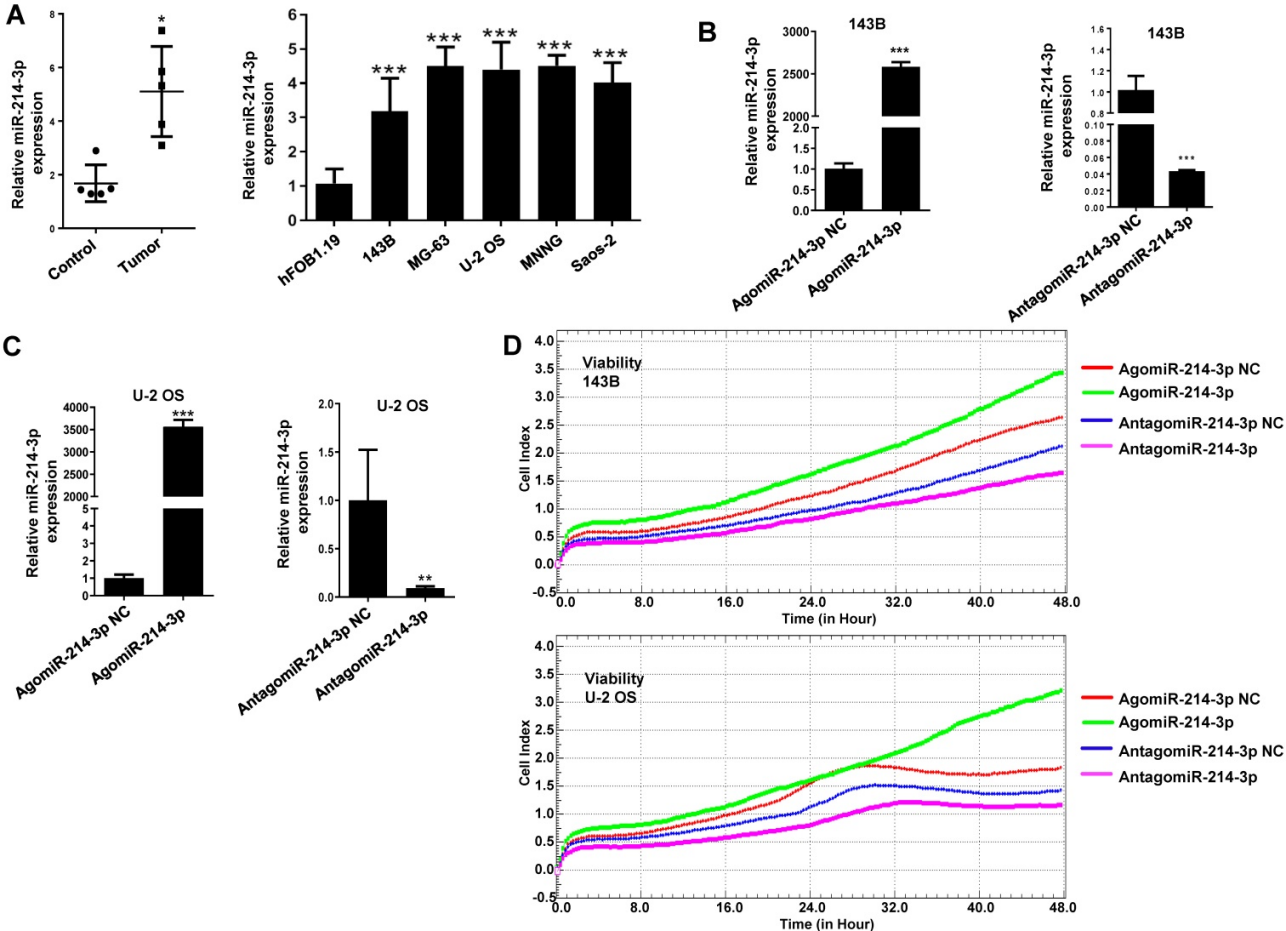

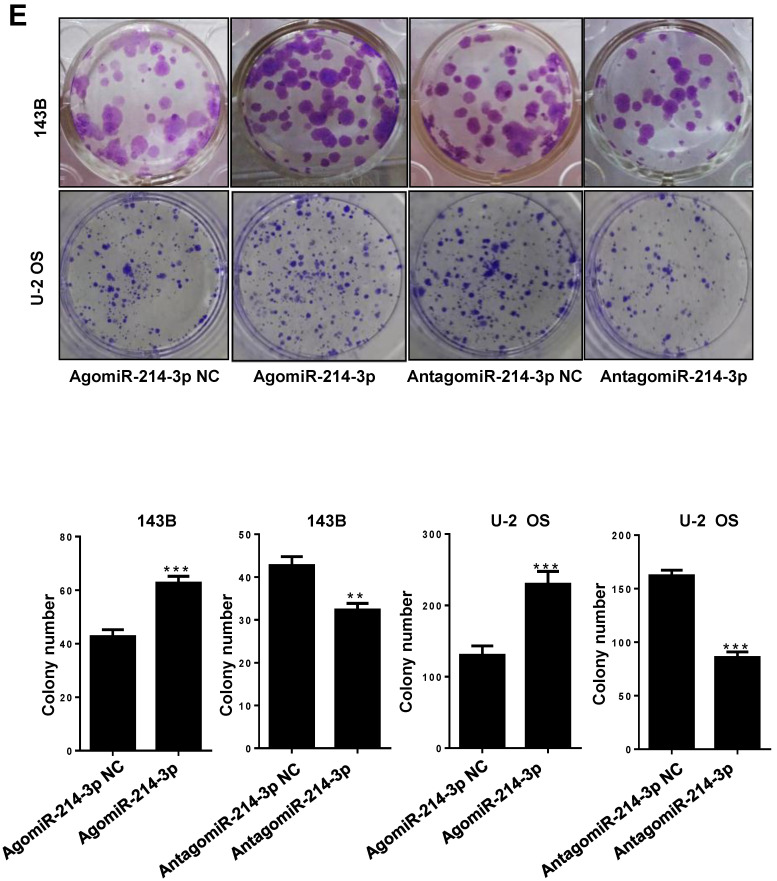

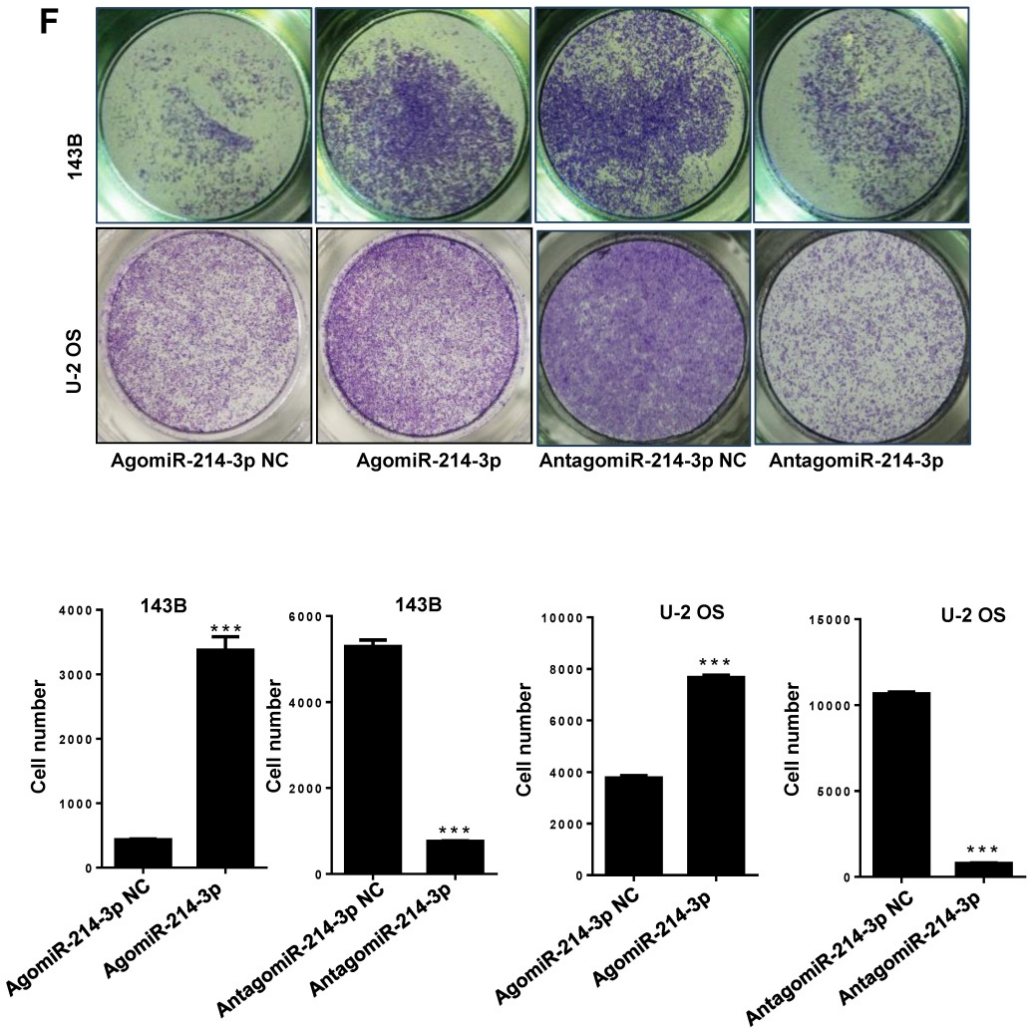

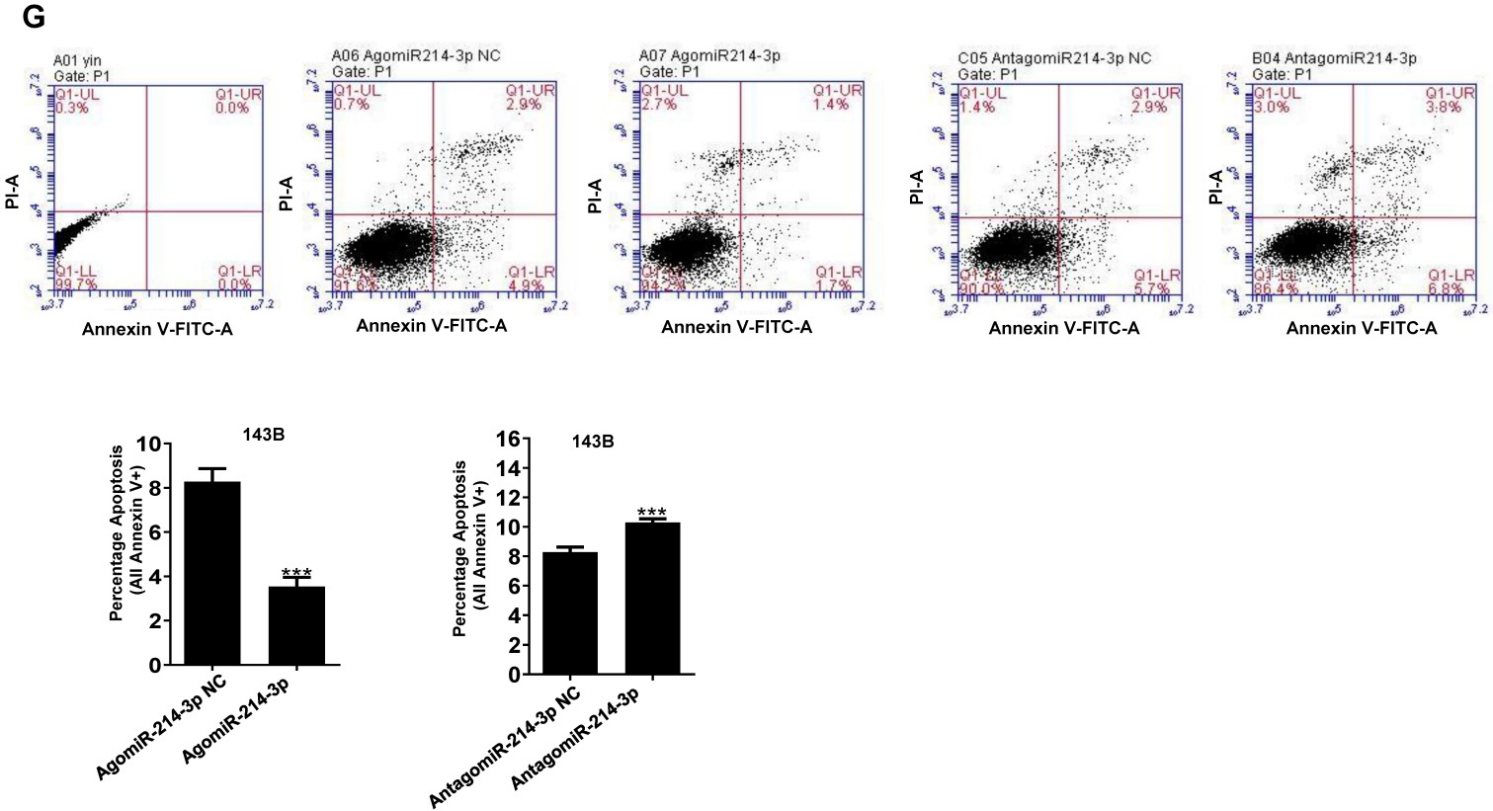
** Up-regulated miR-214-3p identified in human osteosarcoma cells and tissues promotes proliferation and migration, and inhibits apoptosis of osteosarcoma cells.** miR-214-3p expression in paired osteosarcoma and paracancerous normal tissues from 5 osteosarcoma patients (**A,** left panel), in the osteosarcoma cells (143B, U-2 OS, Saos-2, MG-63 and MNNG) and the normal human osteoblast hFOB1.19 cells (**A,** right panel) determined by RT-qPCR assay. RT-qPCR analysis validated successfully over-expressed (AgomiR-214-3p) or silenced (AntagomiR-214-3p) miR-214-3p in 143B **(B)** and U-2 OS **(C)** cells, following phenotypes were then evaluated: **(D)** viability by xCELLigence RTCA DP system monitor, **(E)** self-renew ability with colony formation assay, **(F)** migration by Transwell assay and **(G)** apoptosis by flow cytometric assay. *p < 0.05, **p < 0.01, ***p < 0.001.

For exploring biological function of miR-214-3p in osteosarcoma development, miR-214-3p expression profile was first analyzed in 5 pairs of human osteosarcoma (Tumor) and normal paracancerous (Control) tissues using RT-qPCR, which was identified to be up-regulated significantly in the human osteosarcoma tissues (Fig. 1A, left panel). Furthermore, this finding was validated by evaluating miR-214-3p expression in 5 different human osteosarcoma cell lines (143B, Saos-2, MG-63, U-2 OS and MNNG) and normal human osteoblast hFOB1.19 cells (Fig. 1A, right panel). Taken together, findings above supported the concept of miR-214-3p acting as an oncogenenic factor in osteosarcoma.

### miR-214-3p promotes osteosarcoma progression *in vitro*

**Figure 2:**
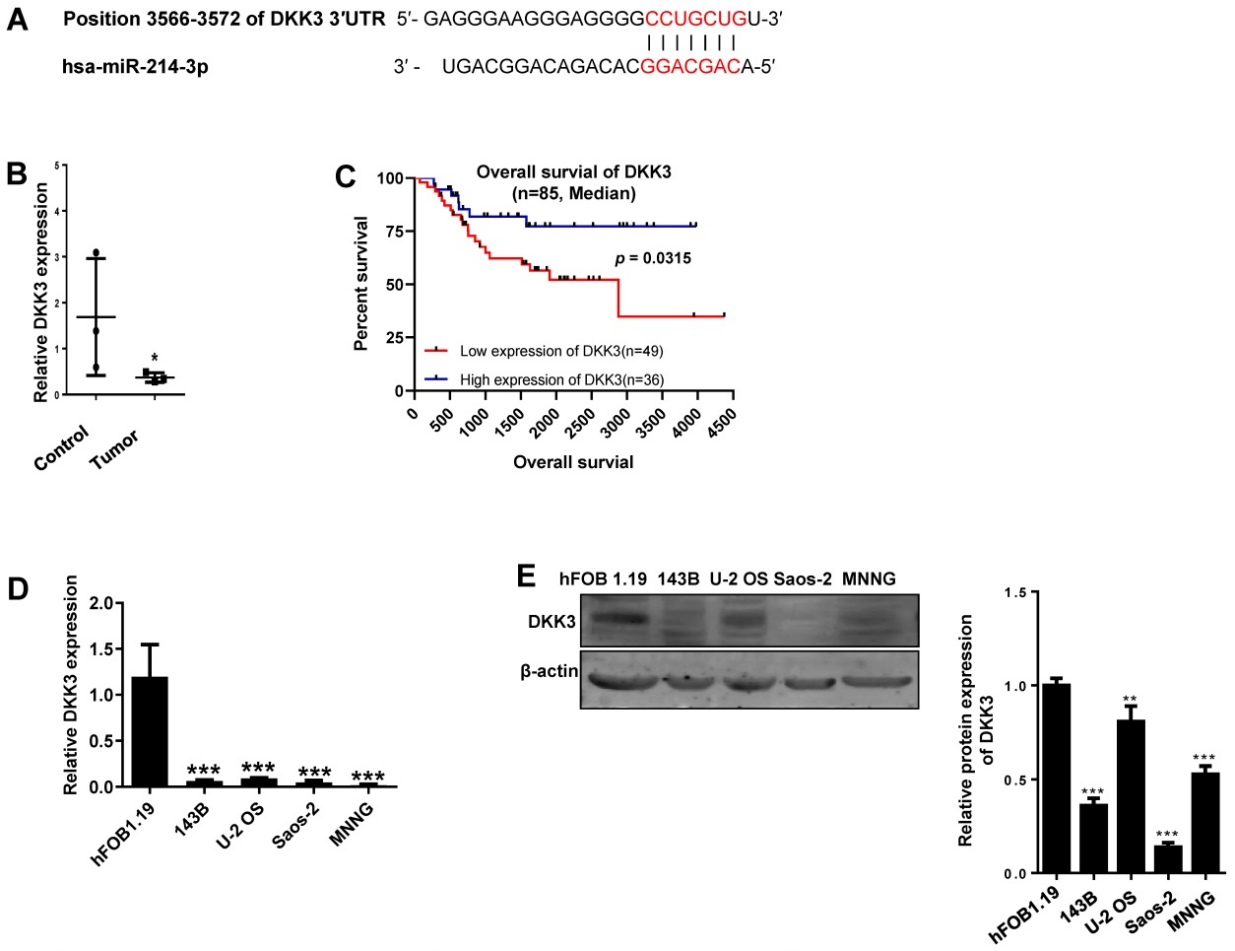

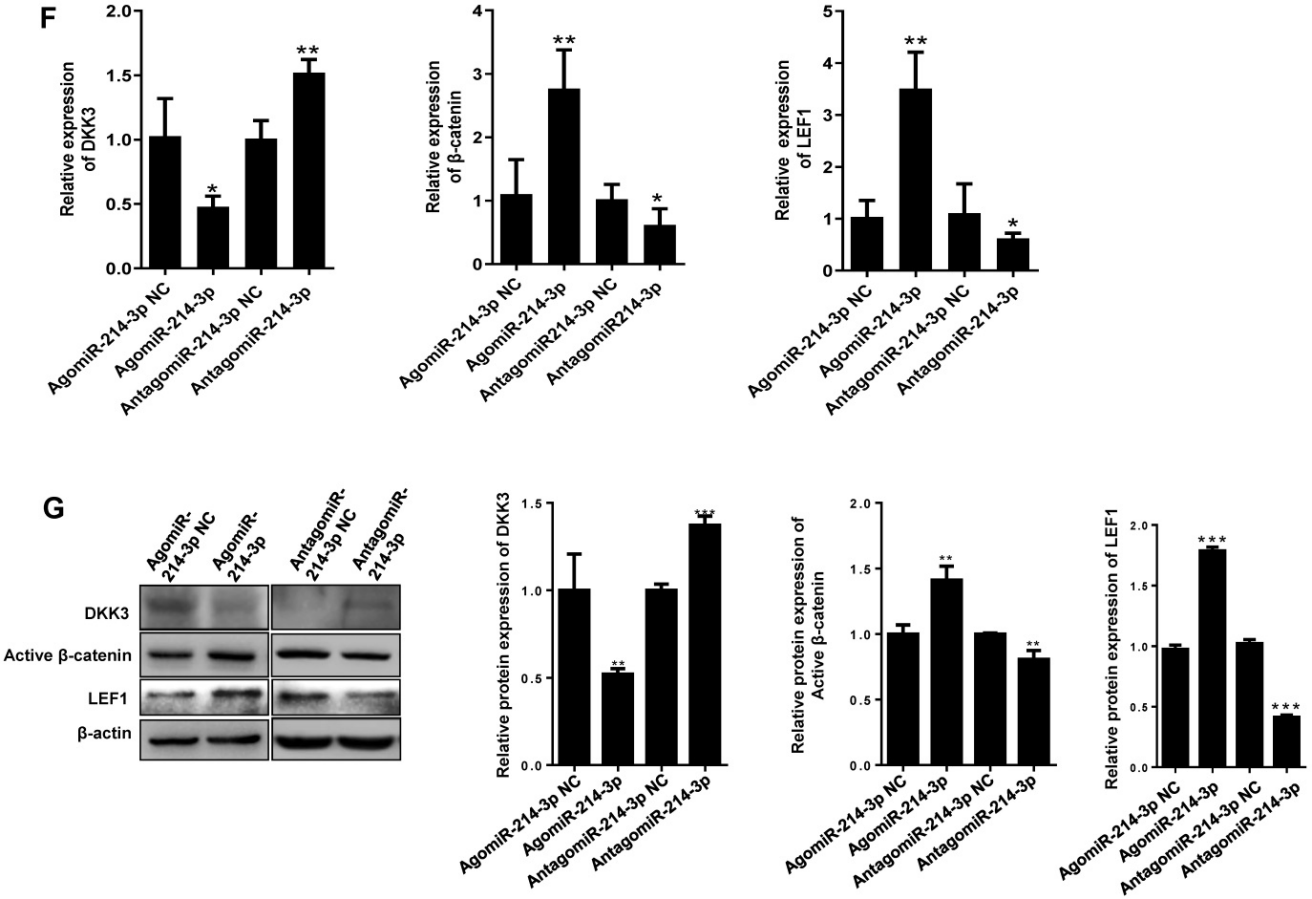
** miR-214-3p promotes proliferation and metastasis of osteosarcoma cells by degrading DKK3 to activate Wnt/β-catenin signaling. (A)** Diagram of potential complementary sequence of miR-214-3p in DKK3 3 -'UTR (http://www.targetscan.org/). **(B)** Expressions of DKK3 mRNA in paired osteosarcoma (Tumor) and paracancerous normal tissues (Control) from 3 patients detected by RT-qPCR. **(C)** Overall survival curves of osteosarcoma patients with Log-rank (Mantel-Cox) test. Expression levels of DKK3 in osteosarcoma cells (143B, U-2 OS, Saos-2 and MNNG) and human osteoblast cell hFOB1.19 determined by RT-qPCR **(D)** and Western blot **(E). (F)** DKK3, β-catenin and LEF1 expressions in 143B cells after knockdown (AntagomiR-214-3p) or over-expression (AgomiR-214-3p) of miR-214-3p determined with RT-qPCR assay. **(G)** DKK3, active-β-catenin and LEF1 expressions in 143B cells after knockdown (AntagomiR-214-3p) or over-expression (AgomiR-214-3p) of miR-214-3p identified by Western blot assay. *p < 0.05, **p < 0.01, ***p < 0.001.

**Figure 3:**
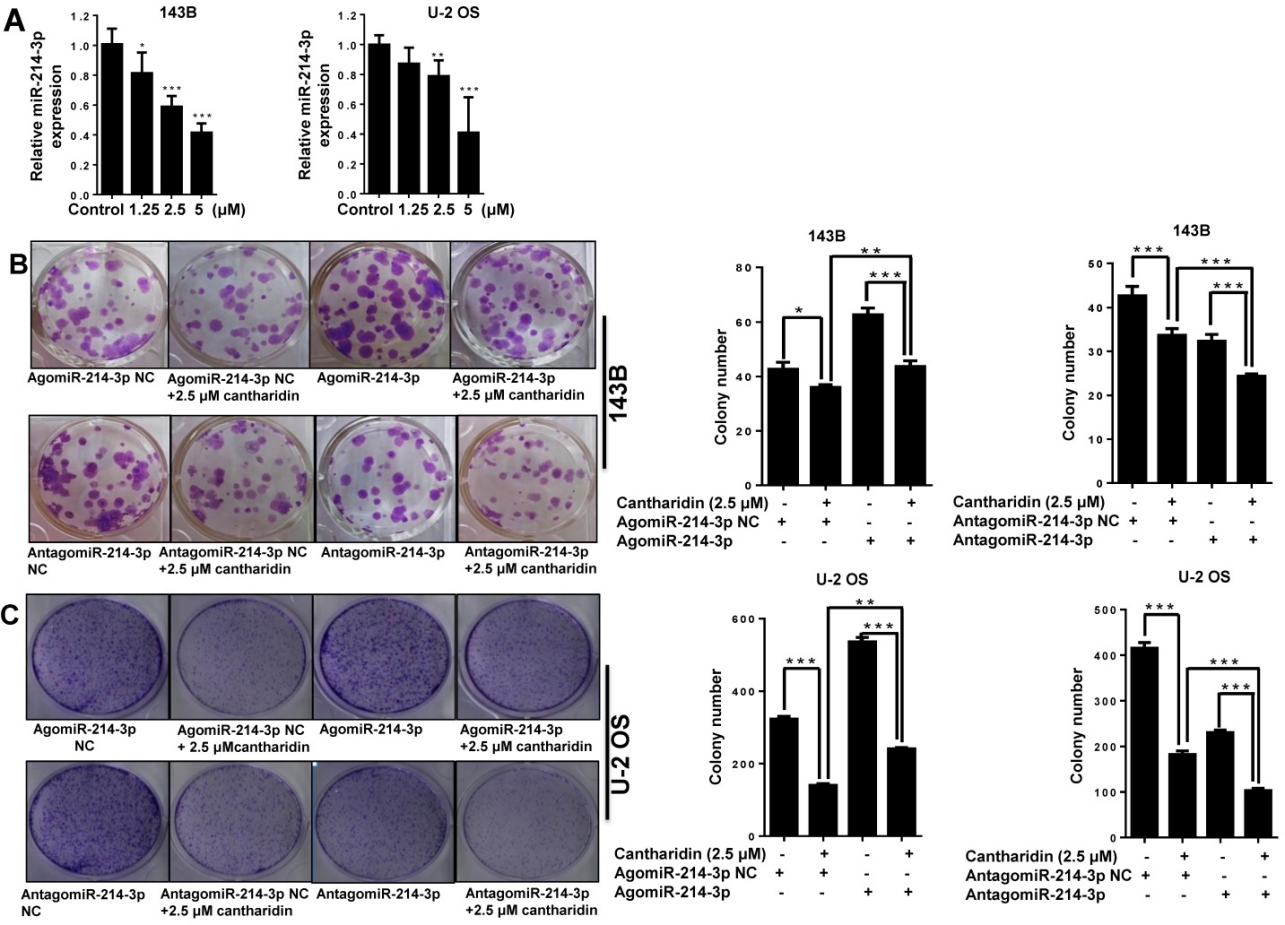

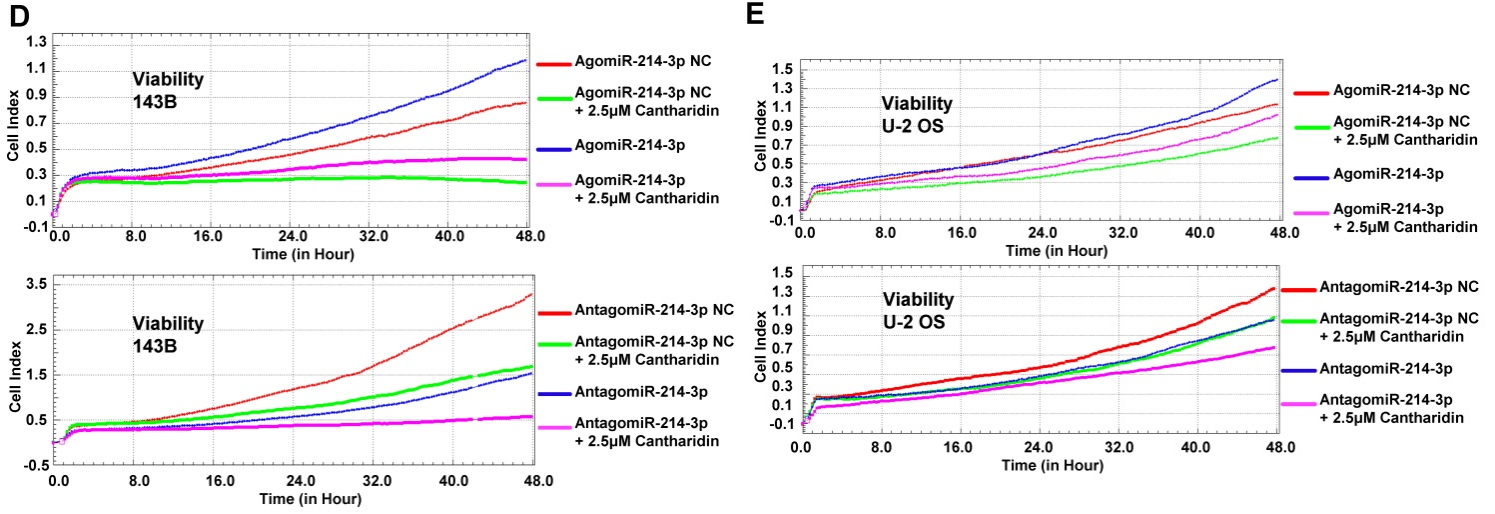

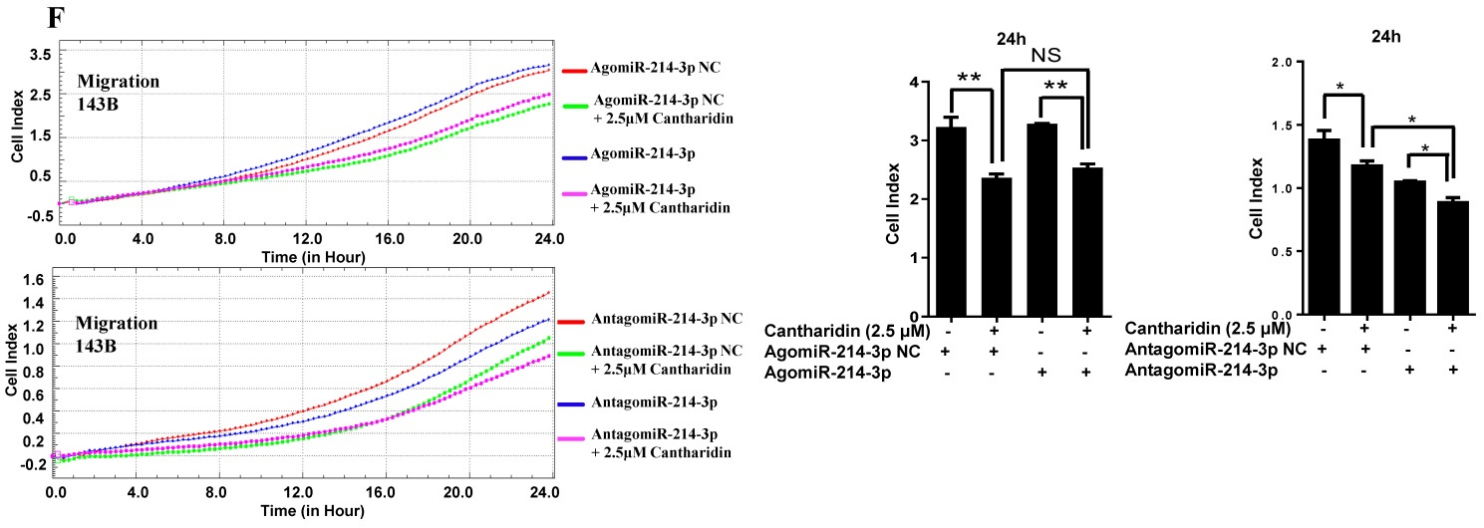

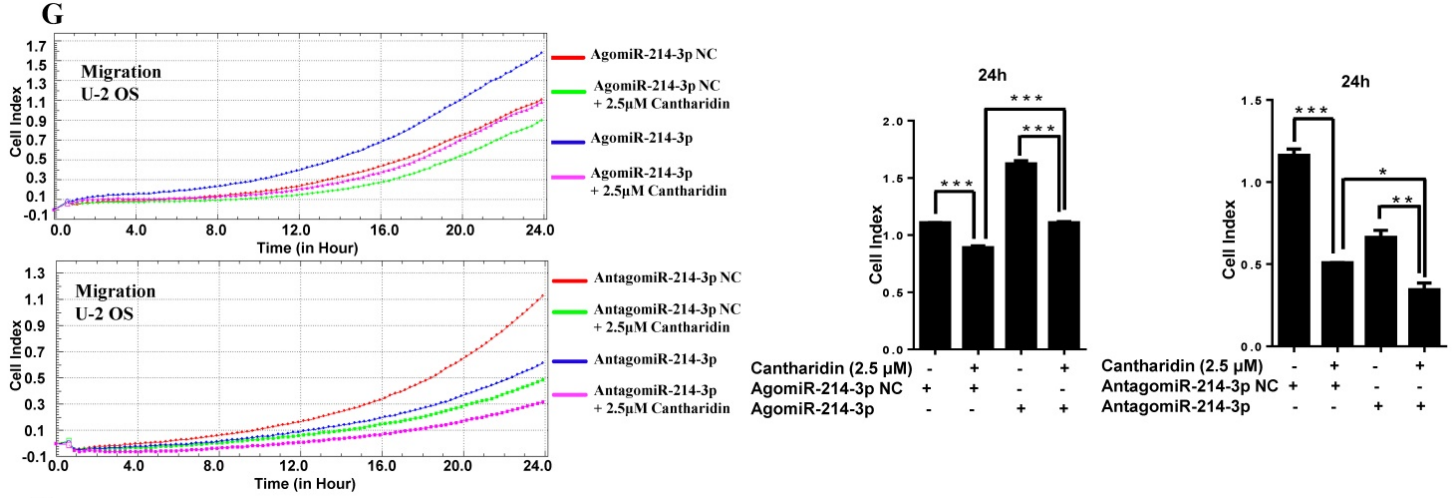

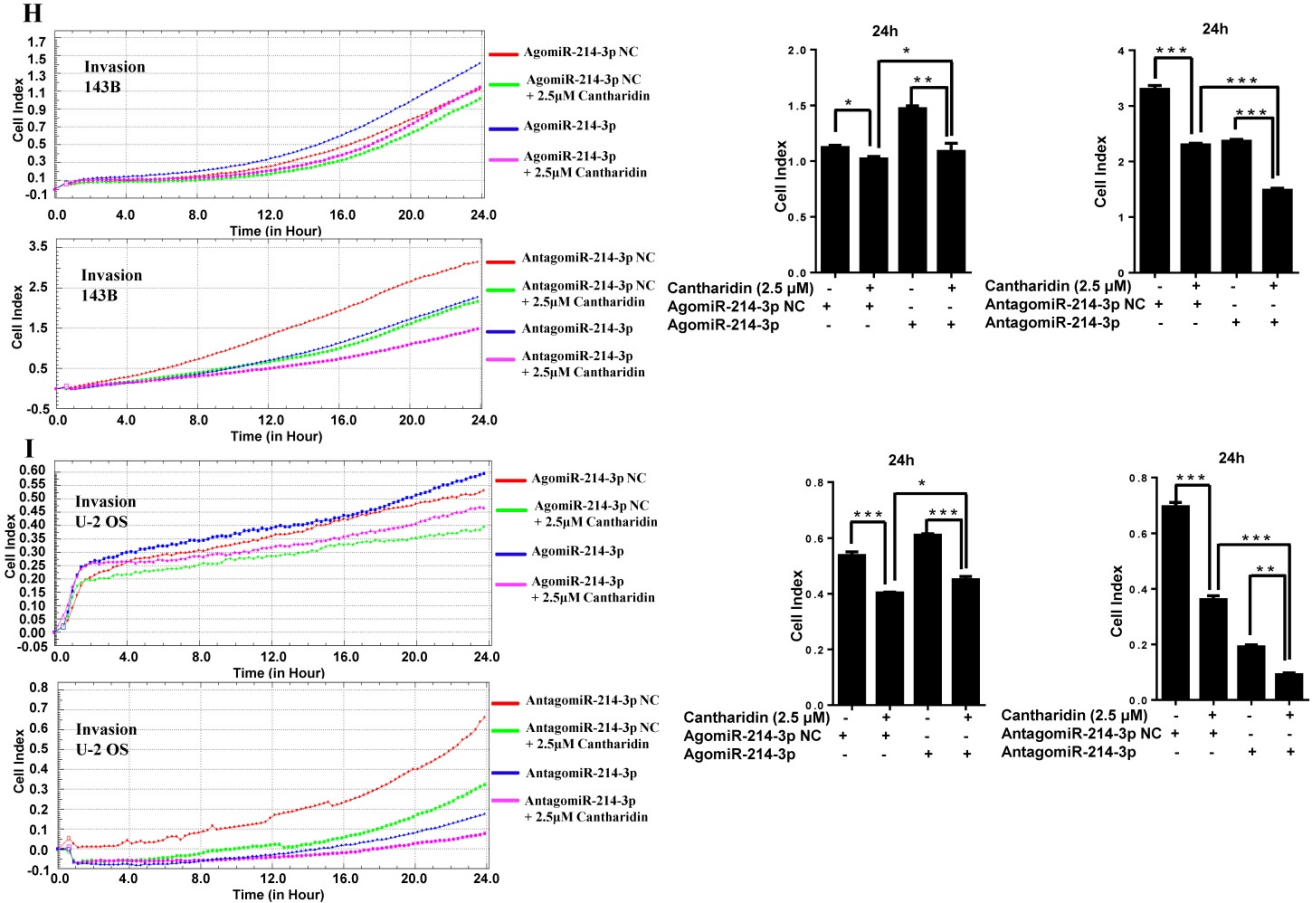
** Cantharidin inhibits proliferation and metastasis of osteosarcoma cells via down-regulating miR-214-3p. (A)** Cantharidin dose dependently inhibited miR-214-3p expression in 143B and U-2 OS cells. To further investigate direct interaction of miR-214-3p in cantharidin inhibited osteosarcoma cell proliferation and metastasis: miR-214-3p was either over-expressed or silenced in 143B and U-2 OS cells before exposure to 0 (DMSO in PBS: 1/10,000 V/V) or 2.5 μM cantharidin, followed by investigating self-renew ability with colony formation assay **(B and C)**; cell viability **(D and E)**, cell migration **(F and G)** and cell invasion **(H and I)** analyzed by xCELLigence RTCA DP system. Statistics assay for Figure 3F-I have been performed by analyzing the data at the time point of 24 h. NS. not statistically significant.*p < 0.05, **p < 0.01, ***p < 0.001.

For further investigating potential function of miR-214-3p in osteosarcoma progression, miR-214-3p expression was either silenced or over-expressed successfully in 143B (Fig. 1B) and U-2 OS (Fig. 1C) cells by transfecting miR-214-3p specific inhibitor (AntagomiR-214-3p) or mimics (AgomiR-214-3p) respectively, and detected by qRT-PCR. Real-time monitor with xCELLigence RTCA DP system indicated that miR-214-3p knockdown inhibited and miR-214-3p over-expression increased viabilities of both 143B and U-2 OS (Fig. 1D) cells, which was confirmed by colony formation assay (Fig. 1E). Migration abilities of 143B and U-2 OS cells were obstructed by miR-214-3p silence, and enhanced by miR-214-3p over-expression evaluated by transwell (Fig.1F), which was further validated by real time monitor with xCELLigence RTCA DP system (Fig. 3F and 3G). Meanwhile, real time monitor with xCELLigence RTCA DP system also showed the similar outcome of miR-214-3p silence or amplification on invasion capacities of 143B (Fig. 3H) and U-2 OS (Fig. 3I) cells. Flow cytometric analysis was conducted to investigate whether outcomes of miR-214-3p silence or amplification on viabilities of 143B cells were associated with the alterations in the apoptosis. The results revealed that miR-214-3p knockdown promoted (Fig. 1G, right panel), and miR-214-3p over-expression inhibited apoptosis (Fig. 1G, left panel) of 143B cells. Collectively, findings above suggested that miR-214-3p promoted oncogenesis and metastasis of osteosarcoma *in vitro*.

### miR-214-3p promotes osteosarcoma progression by targeting the 3ꞌ-UTR of DKK3

**Figure 4:**
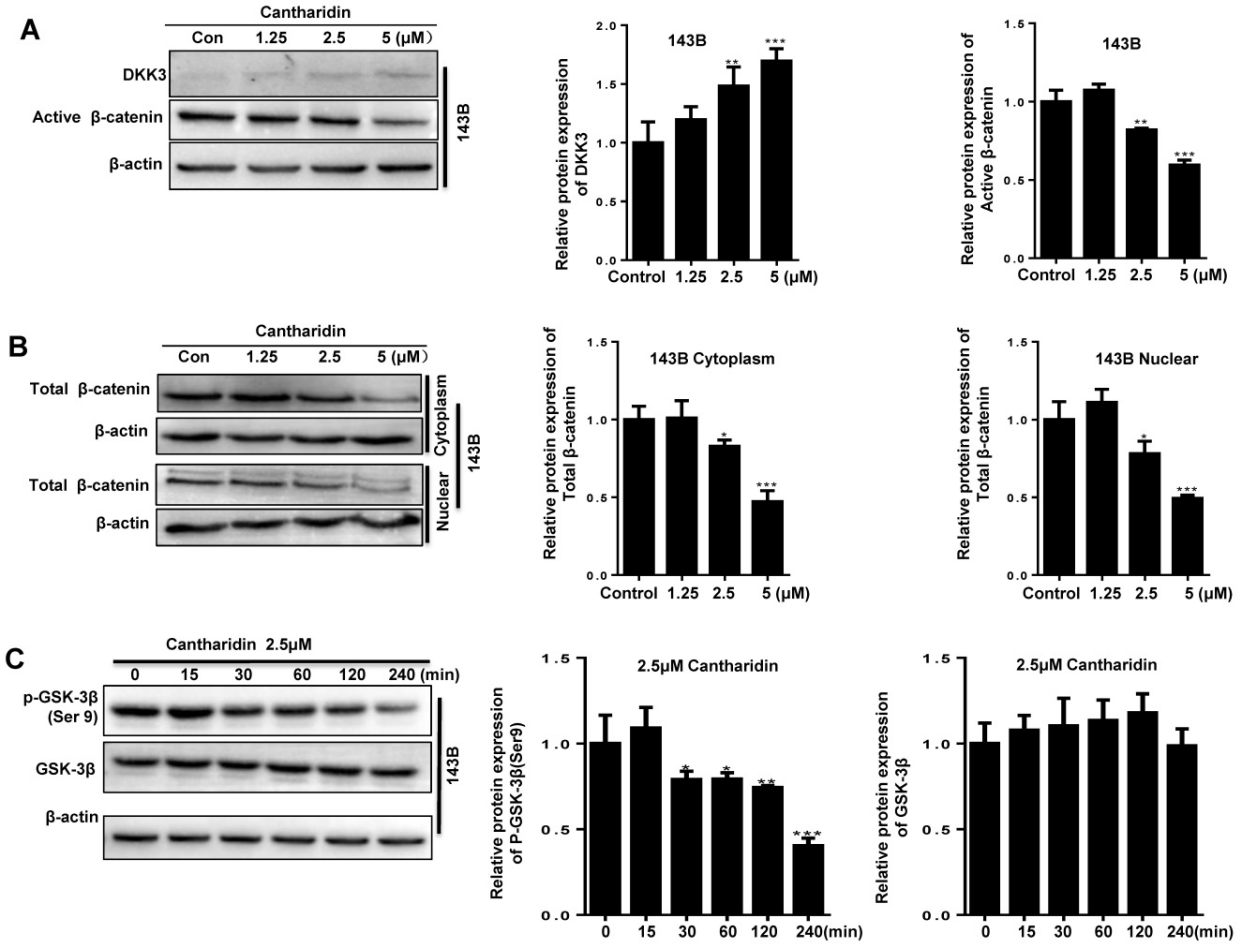

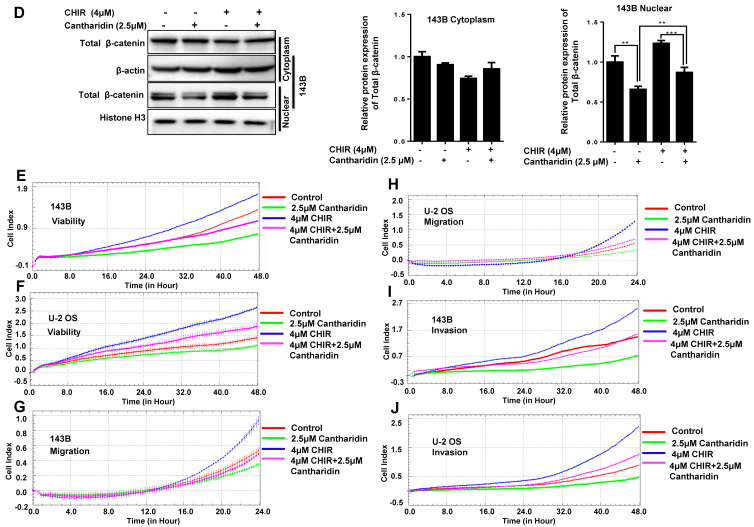

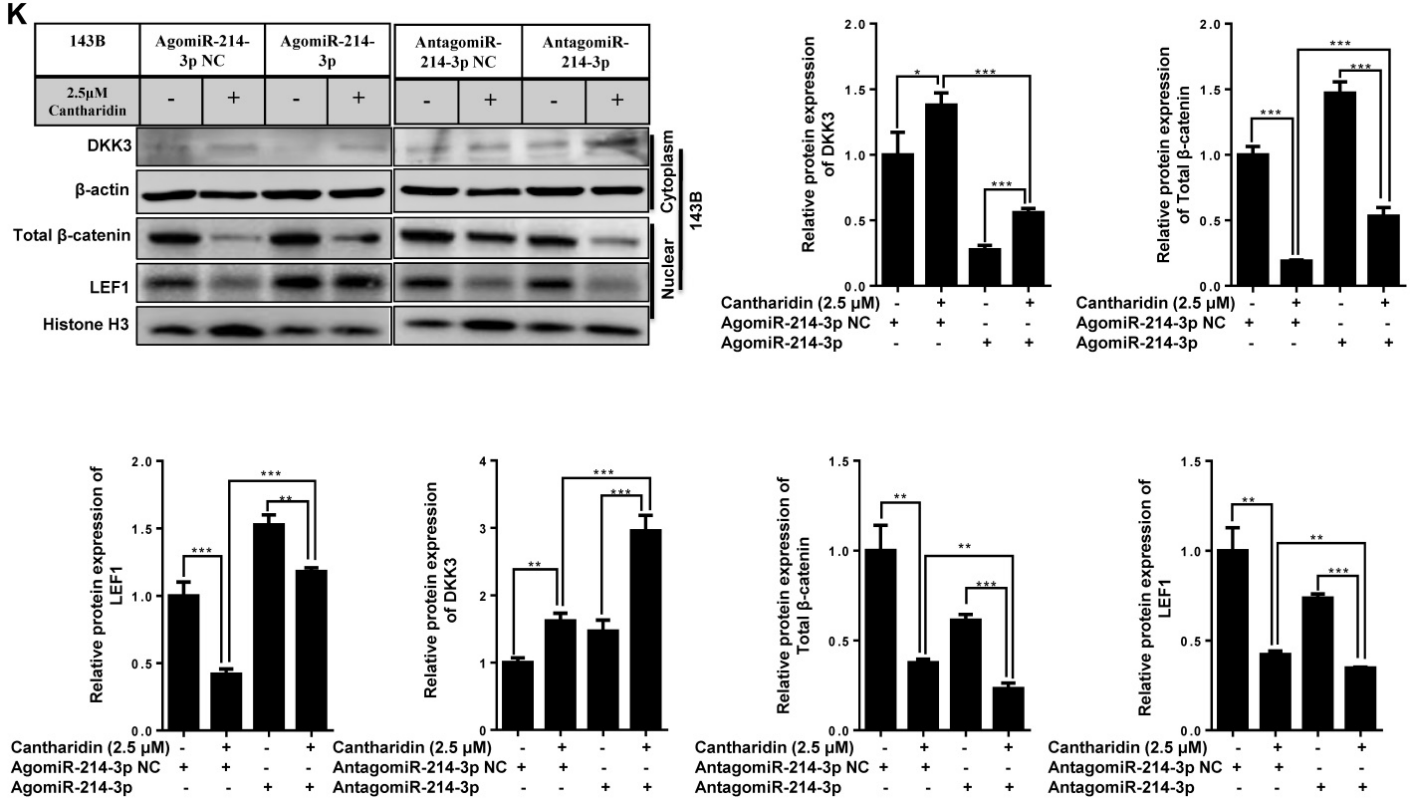
** Cantharidin suppresses osteosarcoma cell progression via specifically targeting miR-214-3p/DKK3/GSK-3β/β-catenin/LEF1 axis.** The 143B cells were respectively exposed to 0, 1.25, 2.5 or 5 μM cantharidin for 24 h, and expressions of DKK3 and active β-catenin in the total proteins were determined by Western blot analysis **(A)**; and expressions of total β-catenin in cytoplasmic and nuclear proteins were further examined with Western blot assay **(B)**.** (C)** p-GSK-3β expression in 143B cells exposed to 2.5 μM cantharidin for different times was determined with Western blot analysis. **(D)**143B cells pretreated with 4 μM CHIR9902 (the specific GSK-3β inhibitor) for 24 h were exposed to 2.5 μM cantharidin for 48 h, the cytoplasmic and nuclear total β-catenin expressions were determined by Western blot assay, the cell viabilities of 143-B **(E)** and U-2 OS **(F)** cells, cell migration abilities of 143-B **(G)** and U-2 OS **(H)** cells, as well as the cell invasion abilities of 143-B **(I)** and U-2 OS **(J)** cells were further recorded with xCELLigence RTCA DP system. **(K)** Cytoplasmic DKK3, as well as the nuclear total β-catenin and LEF1 expressions, were identified with Western blot in 143B cells after being exposed to 2.5 μM cantharidin with or without miR-214-3p over-expression by AgomiR-214-3p or miR-214-3p knockdown by AntagomiR-214-3p. *p < 0.05, **p < 0.01, ***p < 0.001.

Given the importance of Wnt/β-catenin signaling in promoting osteosarcoma progression, the DKK3 acting as an extracellular Wnt/β-catenin inhibitor, as well as a 3′-UTR sequence of DKK3 complementary to the seed sequence of miR-214-3p being identified by targetscan online analysis (http://www.targetscan.org/) (Fig. 2A), we hypothesized that miR-214-3p may degrade DKK3 by binding to its 3'-UTR and achieve the role in promoting the oncogenesis and development of osteosarcoma. To evidence this hypothesis, we first evaluated mRNA expression of DKK3 by RT-qPCR in paired human osteosarcoma (Tumor) and paracancerous normal tissues (Control) from 3 patients (Fig. 2B). The results revealed a significantly decreased DKK3 expression in osteosarcoma tissues. Meanwhile, patients with low DKK3 expression showed a significant decreased overall survival rate compared with patients with high DKK3 expression based on the TARGET database (Fig. 2C, *p* = 0.0315). The decreased DKK3 expression was further confirmed in osteosarcoma cells (143B, U-2 OS, MNNG, and Saos-2) than in hFOB1.19 cells by RT-qPCR (Fig. 2D) and Western blot (Fig. 2E) assays. Expressions of DKK3, β-catenin and LEF1 in 143B cells after knockdown or over-expression of miR-214-3p were further determined. The data revealed that miR-214-3p knockdown caused an up-regulated DKK3, a down-regulated β-catenin and LEF1 mRNA expression, meanwhile, miR-214-3p over-expression caused an down-regulated DKK3, an up-regulated β-catenin and LEF1 mRNA expression (Fig.2F); these findings were further validated by protein expressions of DKK3, active β-catenin and LEF1 in the total proteins (Fig. 2G), as well as total β-catenin and LEF1 in nuclear proteins (Fig. 4K), which showed miR-214-3p over-expression suppressed DKK3 expression and increased active β-catenin in the total proteins, as well as augmented the nuclear β-catenin and LEF1 expression. Therefore, these findings suggested that miR-214-3p promoted activation and β-catenin nuclear translocation by down-regulating DKK3 expression. This further activate translation of LEF1 in nuclear, resulting in activation of Wnt/β-catenin signaling in 143B cells, thus to promote oncogenesis and metastasis of osteosarcoma.

### Cantharidin inhibits osteosarcoma cell proliferation and metastasis via down-regulation of miR-214-3p

To determine whether miR-214-3p involves the anti-osteosarcoma effect of cantharidin, we measured the miR-214-3p expressions in 143B and U-2 OS cells with RT-qPCR after cantharidin exposure for 24 h at concentrations of 1.25, 2.5 or 5 μM. Cantharidin was found to dose-dependently (0-5 μM) inhibit miR-214-3p expressions in 143B and U-2 OS cells (Fig. 3A).

Furthermore, specific AgomiR, AgomiR NC, AntagomiR and AntagomiR NC for miR-214-3p were respectively transfected into 143B and U-2 OS osteosarcoma cells via RNAiMAX before 2.5 μM cantharidin treatment, then the self-renew abilities by colony formation assay (Fig. 3B and 3C); cell viabilities (Fig. 3D and 3E), cell migration (Fig. 3F and 3G) and invasion (Fig. 3H and 3I) abilities using xCELLigence RTCA DP system were investigated, which demonstrated that 2.5 μM cantharidin significantly reduced the colony formation, cell viability, cell migration and invasion in both the 143B and U-2 OS osteosarcoma cells (AgomiR-214-3p NC+2.5 μM cantharidin or AntagomiR-214-3p NC+2.5 μM cantharidin) compared with the cells without cantharidin treatment (AgomiR-214-3p NC or AntagomiR-214-3p NC). However, these inhibitory effects were sensitized with miR-214-3p knockdown (AntagomiR-214-3p + 2.5 μM cantharidin) and partly rescued with miR-214-3p over-expression (AgomiR-214-3p + 2.5 μM cantharidin). Altogether, these findings suggested that cantharidin inhibited proliferation and metastasis of osteosarcoma cells via specifically down-regulating the miR-214-3p expression.

### Cantharidin inhibits osteosarcoma progression by specifically targeting the miR-214-3p/DKK3/GSK-3β/Wnt/β-catenin/LEF1 axis

To find out whether cantharidin inhibited osteosarcoma progression via down-regulating miR-214-3p expression was achieved by specifically targeting the DKK3 expression, thus to decrease β-catenin nuclear translocation and cause an reduced Wnt/β-catenin signaling activity in osteosarcoma cells, we firstly investigated outcome of cantharidin on DKK3/GSK-3β/Wnt/β-catenin signaling. The results revealed that cantharidin treatment dose-dependently (0-5 μM) increased the DKK3 expression level, while decreased the active (non-phospho) β-catenin expression level in the total proteins of 143B cells (Fig. 4A); and down-regulated the total β-catenin (Fig. 4B) and LEF1 (Fig. 4K; AgomiR-214-3p NC+2.5 μM cantharidin or AntagomiR-214-3p NC + 2.5 μM cantharidin) in the nuclear proteins of 143B cells; meanwhile, p-GSK-3β expression in the 143B cells was found to be time-dependently (0-240 min) inhibited by 2.5 μM cantharidin (Fig. 4C). Moreover, to validate the anti-osteosarcoma effect is achieved specifically via cantharidin-caused Wnt/β-catenin signaling inactivation; we pretreated 143B cells using specific GSK-3β inhibitor (CHIR99021) before being challenged using 2.5 μM cantharidin. The results showed that CHIR99021 reversed cantharidin related inhibition of Wnt/β-catenin signaling activity and anti-osteosarcoma effects in 143B cells, evidenced by partly rescued total β-catenin expression in the nuclear proteins (Fig. 4D), cell viabilities (Fig. 4E-F), cell migration (Fig. 4G-H) and invasion (4I-J) abilities. Our findings demonstrated that cantharidin inhibited osteosarcoma survival partially via inactivating Wnt/β-catenin signaling. Moreover, the protein expressions of DKK3 in the total proteins, as well as the nuclear total β-catenin and LEF1 of 143B cells, were detected after cantharidin exposure with or without co-transfecting AntagomiR-214-3p (miR-214-3p silence) or AgomiR-214-3p (miR-214-3p over-expression) with Western blot; our results disclosed that cantharidin treatment promoted DKK3 expression in the total proteins, inhibited total β-catenin and LEF1 in the nuclear proteins of 143B cells, which were partially reversed via miR-214-3p over-expression, while enhanced via miR-214-3p silence (Fig. 4K). Together, these data showed that DKK3 was up-regulated when the miR-214-3p was inhibited by the cantharidin in the osteosarcoma cells; the up-regulated DKK3 prevented the GSK-3β from being phosphorylated, which further inhibited the β-catenin activation, nuclear translocation, and the downstream LEF1 translation, leading to an inhibited Wnt/β-catenin signaling activity in osteosarcoma cells, therefore, confirmed direct contribution of miR-214-3p/DKK3/GSK-3β/β-catenin/LEF1 axis in cantharidin suppressed osteosarcoma progression.

### Cantharidin arrests osteosarcoma cell cycle at M phase and triggers the apoptosis

To further explore whether apoptosis induction and cell cycle arrest also involve in anti-proliferation activity of cantharidin in osteosarcoma cells, the 143B and U-2 OS cells were exposed to diverse concentrations of cantharidin (1.25, 2.5 or 5 μM) for 24 h. Then apoptosis rate and cell cycle distribution were estimated with flow cytometric analysis after Annexin V-FITC/PI and PI staining, respectively.

**Figure 5:**
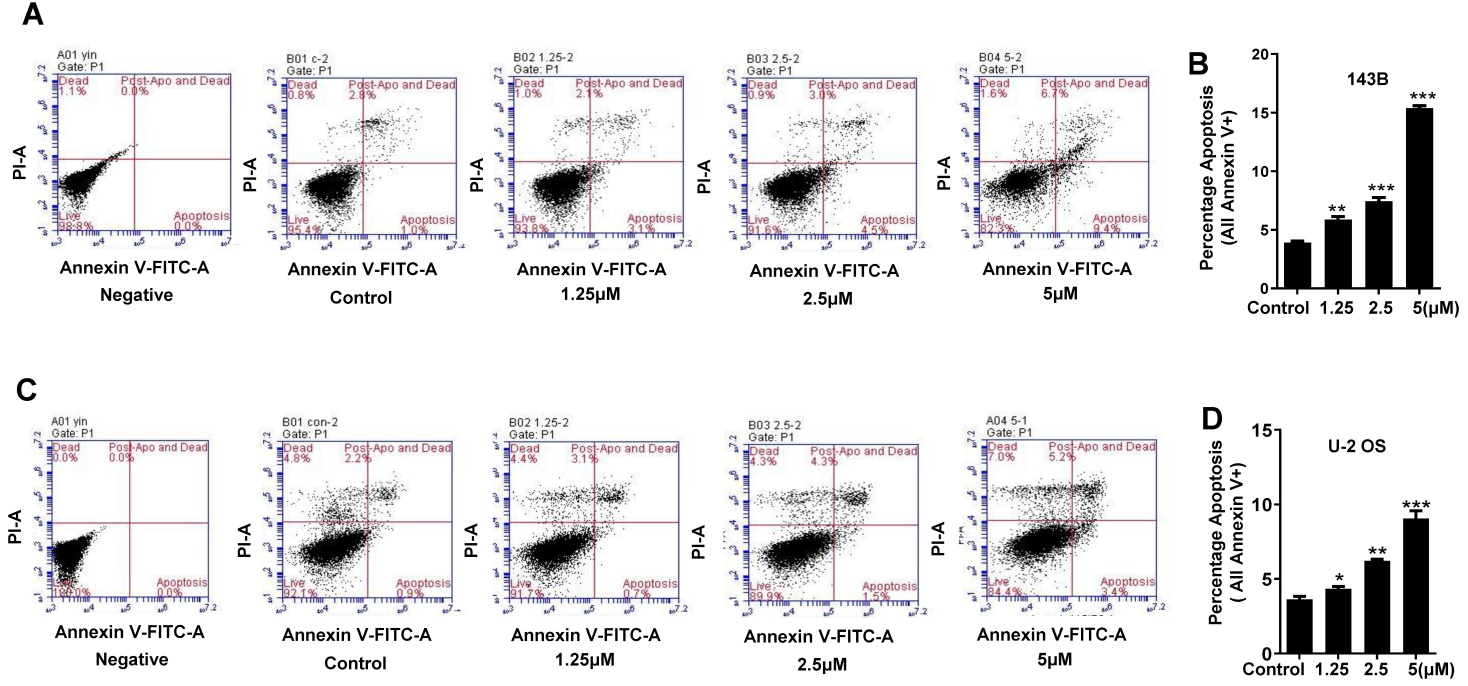

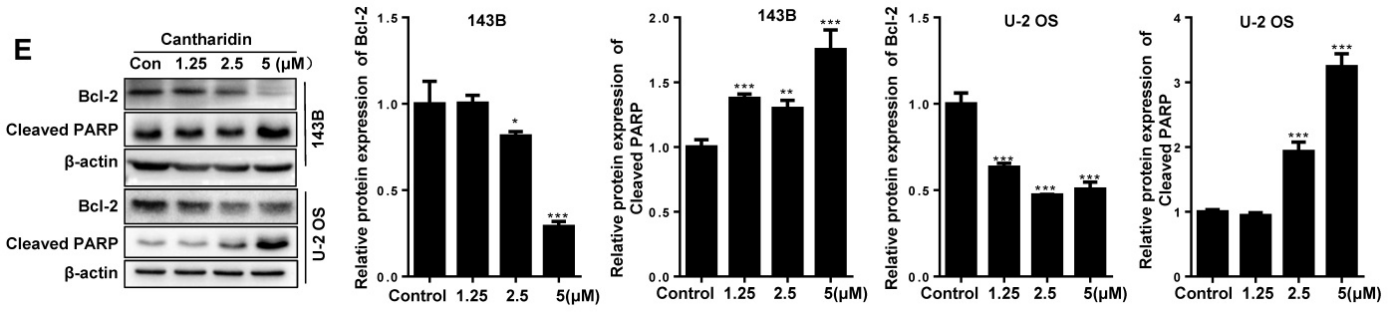

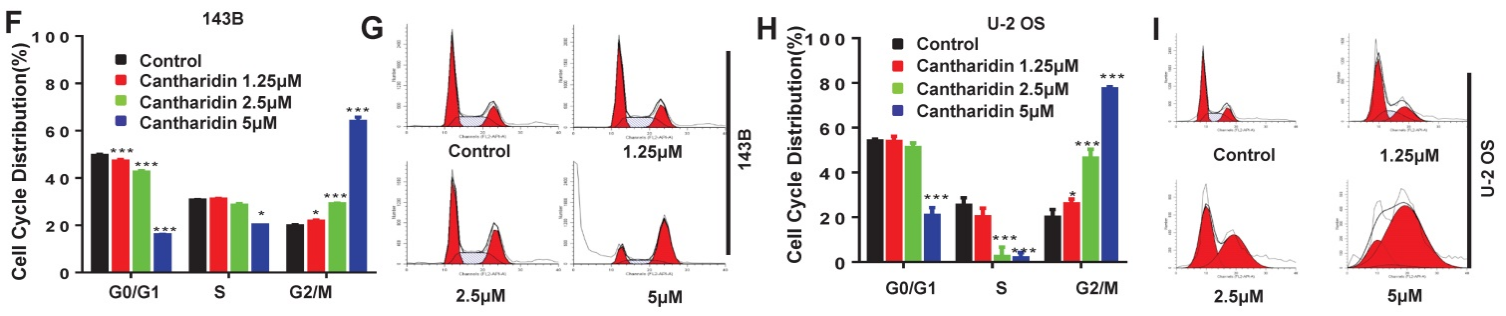

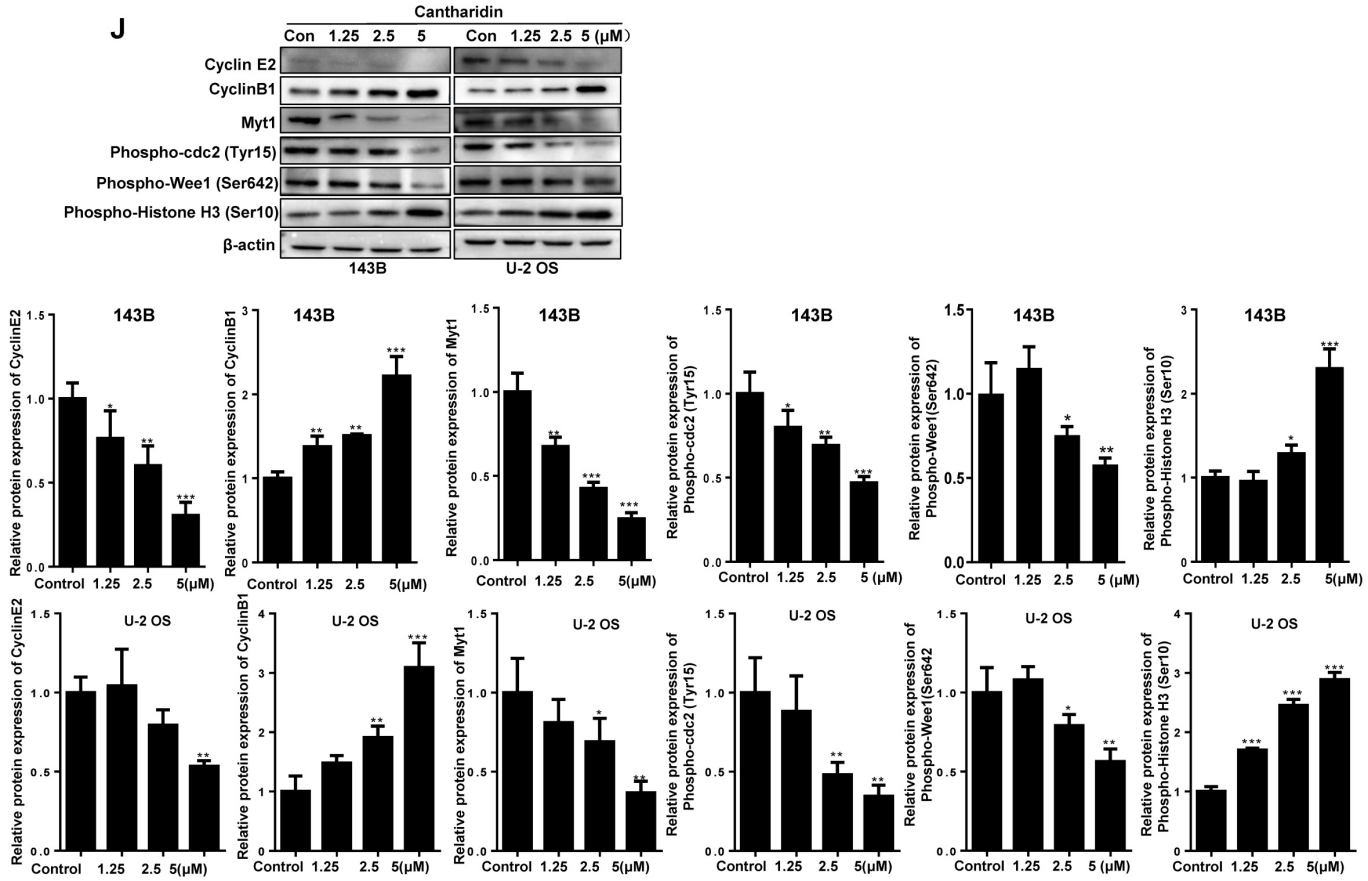
** Cantharidin promotes cell cycle arrest and apoptosis in 143B and U-2 OS cells measured with flow cytometer and Western blot. (A-E)** Dose-dependently (0, 1.25, 2.5 and 5 μM) triggered apoptosis of 143B and U-2 OS cells: **(A)** apoptosis rate of 143B cells; **(B)** representative diagrams of apoptotic 143B cells; **(C)** apoptotic rate of U-2 OS cells; **(D)** representative diagrams of apoptotic U-2 OS cells; **(E)** expressions of key proteins related to apoptosis. **(F-J)** Cell cycles of 143B and U-2 OS cells were arrested at M phase: **(F and G)** specific phase distribution and representative diagrams through cell cycle of 143B cells; **(H and I)** specific phase distribution and representative diagrams through cell cycle of U-2 OS cells; **(J)** expressions of cell cycle related key proteins identified by Western blot assay. β-actin served as a loading control for Western blot assay. *p < 0.05, **p < 0.01, ***p < 0.001.

Our data revealed that cantharidin dose-dependently (0-5 μM) triggered the apoptosis of 143B and U-2 OS cells (Fig. 5A-5D), which was further confirmed by amplified apoptotic effector protein (Cleaved PARP) expression and reduced anti-apoptotic protein (Bcl-2) expression in both 143B and U-2 OS cells with cantharidin intervention (Fig. 5E). Meanwhile, our data also found that cantharidin arrested the 143B and the U-2 OS cells at G2/M phase, accompanied by dose-dependent decrease of cell numbers in G0/G1 and S phases (Fig. 5F-5I). Furthermore, this was validated by reduced representative protein (cyclin E2) expression in G1/S phase, and increased representative protein (cyclin B1) expression in G2/M phase (Fig. 5J). To further determine whether cell cycle was arrested in G2 phase or M phase, the expressions of representative proteins in G2 phase and M phase were identified, respectively. Our data indicated that expressions of representative proteins in G2 phase [Myt1, phospho-cdc2 (Tyr15) and phospho-Wee1 (Ser642)] were decreased, and expression of representative protein in M phase [phospho-Histone H3 (Ser10)] was increased (Fig. 5J) in 143B and U-2 OS cells.

Altogether, these data revealed that cantharidin treatment effectively stimulated cell cycle arrest at M phase and apoptosis of human osteosarcoma cells.

### Cantharidin inhibits human osteosarcoma cell migration and invasiveness by suppressing epithelial-mesenchymal transition (EMT)

**Figure 6:**
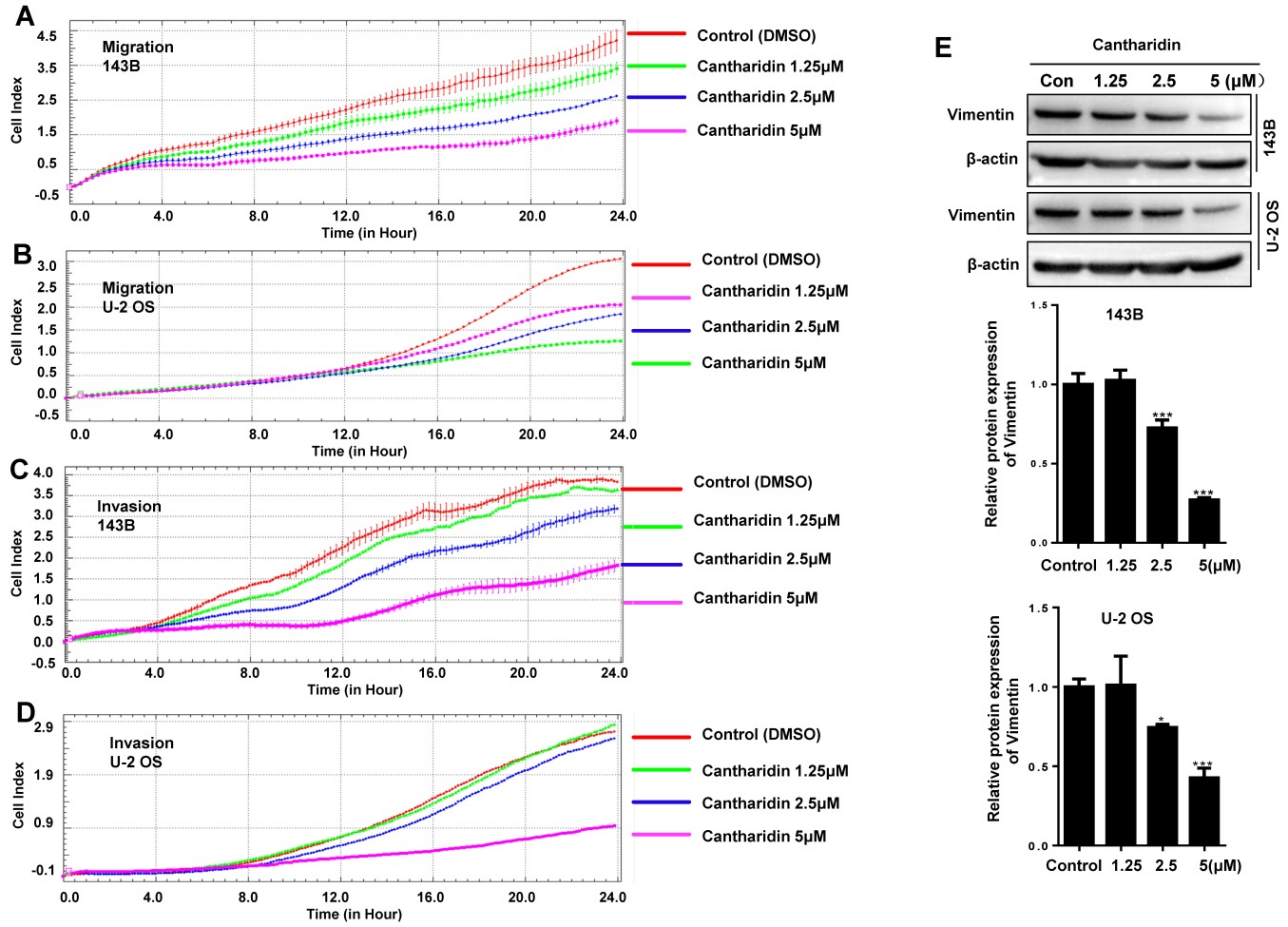
** Cantharidin inhibits osteosarcoma cell migration and invasion *in vitro* measured with Western blot assay and xCELLigence RTCA DP system.** 143B and U-2 OS cells were respectively exposed to 0, 1.25, 2.5 or 5μM cantharidin: **(A)** 143B cell migration; **(B)** U-2 OS cell migration; **(C)** 143B cell invasion; **(D)** U-2 OS cell invasion; **(E)** Western blot detected expression of EMT-related factor Vimentin with β-actin as a loading control. *p < 0.05, **p < 0.01, ***p < 0.001.

To investigate whether cantharidin affects the metastasis of human osteosarcoma cells, 143B and U-2 OS cell migration abilities were continuously monitored with an xCELLigence RTCA DP system, which indicated that the migration abilities of 143B (Fig. 6A) and U-2 OS (Fig. 6B) cells were dose-dependently (0-5 μM) and time-dependently (0-24 h) inhibited by the cantharidin.

To further explore if cantharidin affects the invasive abilities of human osteosarcoma cells, we monitored the real-time invasiveness of cantharidin treated 143B and U-2 OS cells using xCELLigence RTCA DP system with a matrigel-coated upper chamber in the CIM-Plate. The results showed that cantharidin dose-dependently (0-5 μM) and time-dependently (0-48 h) suppressed the invasiveness of 143B (Fig. 6C) and U-2 OS (Fig. 6D) cells versus control cells exposed to DMSO in PBS only. Expression of Vimentin, a specific mesenchymal phenotype molecular marker, in the total cell lysates from 143B and U-2 OS cells after being exposed to cantharidin was further analyzed respectively by Western blot assay. This result showed that cantharidin dose-dependently (0-5 μM) down-regulated the Vimentin expression levels in both the 143B and U-2 OS cells (Fig. 6E). Collectively, these data suggested that cantharidin may suppress human osteosarcoma cell migration and invasion by reversing EMT* in vitro*.

### Cantharidin inhibits human osteosarcoma cell proliferation both *in vivo* and *in vitro*

**Figure 7:**
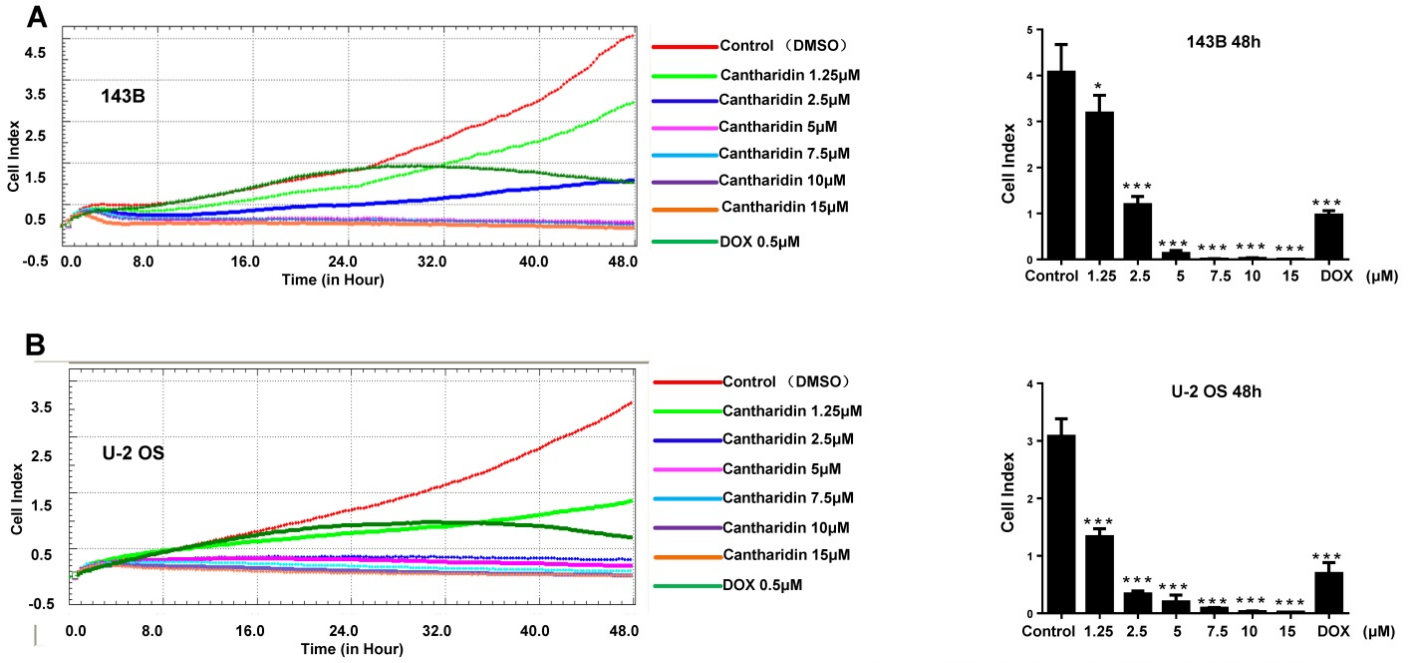

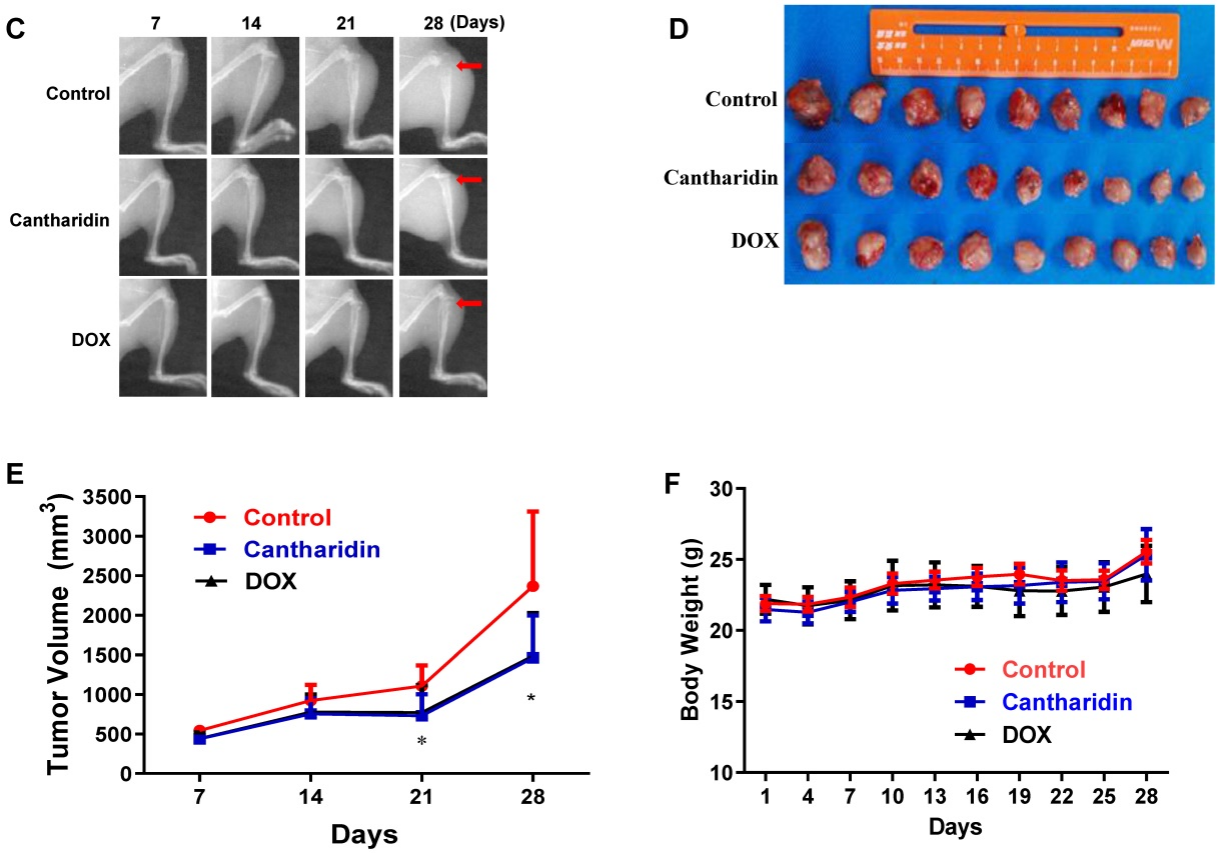
** Cantharidin inhibits osteosarcoma growth *in vivo* and *in vitro*.** The viabilities of 143B **(A)** and U-2 OS **(B)** cells after cantharidin treatment at the doses of 0-15 μM for 0-48h were measured by an xCELLigence RTCA DP system. Osteosarcoma growth was suppressed after cantharidin treatment for 28 days in xenograft-bearing nude mice with 143B cells injection into the tibia(n=9): **(C)** orthotopic tumor growth was dynamically monitored once a week with X-ray using the Image Station In-Vivo FX system, **(D)** images of the isolated xenograft tumors, **(E)** tumor volumes, and **(F)** body weights of mice. Mice in control group were intervened with DMSO in PBS (1/10,000 V/V). DOX (1 mg/kg·body weight) served as positive control. *p < 0.05, **p < 0.01. DOX, doxorubicin.

To explore effect of cantharidin on osteosarcoma cell proliferation, viable cell numbers were continuously recorded by xCELLigence RTCA DP system for 48 h when 143B and U-2 OS cells were challenged with diverse concentrations of cantharidin (1.25, 2.5, 5, 7.5, 10 or 15 μM), using DMSO (1/10,000 V/V) and DOX (0.5 μM) as negative and positive controls, respectively. Results showed that cantharidin dose- and time-dependently inhibited the viabilities of 143B (Fig. 7A) and U-2 OS (Fig. 7B) cells.

Moreover, to investigate whether cantharidin inhibit osteosarcoma growth* in vivo*, from the second day after 143B cells were injected to left tibia of each nude mouse, therapeutic reagents were intraperitoneally injected once a day for 28 days as follows: same volumes of cantharidin (2.5 mg/kg·body weight) for cantharidin group, DMSO (1/10,000 V/V) for control group, and DOX (1 mg/kg·body weight) for positive control group, respectively. Tumor masses were isolated and compared between control (DMSO in saline) and cantharidin groups after mice were euthanized. Cantharidin was found to significantly inhibit osteosarcoma lesion in the tibia of nude mice detected by X-ray (Fig. 7C); as we can see, the xenograft-bearing legs in the control group showed significant invasive tumors which damaged the bone marrow barrier and caused the bone destruction; however, the xenograft-bearing legs of the cantharidin treated mice showed less architecture destruction with limited tumors and intact bone infrastructure. This was further confirmed by the significantly suppressed tumor growth evidenced by the deceased size (Fig. 7D) and tumor volume (Fig. 7E) of the isolated tumor masses. Meanwhile, mouse body weights among groups showed no difference (Fig. 7F). Overall, these data confirmed the anti-proliferation activity of cantharidin in osteosarcoma cells both *in vivo* and *in vitro*.

## Discussion

No fundamental improvements have been achieved in the clinical outcomes and therapeutic strategies for osteosarcoma patients over the past decades 2; consequently, it is crucial to develop novel treatments for improving prognosis of osteosarcoma patients 19. Studies have evidenced that dysregulation of miRNAs is related to oncogenesis, progression and poor prognosis of many malignant tumor types, including osteosarcoma 23, 24. Here, we revealed a significant up-regulation of miR-214-3p in human osteosarcoma tissues and cells. miR-214-3p knockdown inhibited, while over-expression increased osteosarcoma cell proliferation and metastasis; meanwhile, miR-214-3p knockdown induced, while over-expression inhibited apoptosis of osteosarcoma cells, suggesting the carcinogenetic function of miR-214-3p in osteosarcoma and its significance as a prognostic prediction biomarker in osteosarcoma patients.

The miRNAs mainly inhibit translation or promote target mRNA degradation by binding to a complementary sequence (seed sequence) within their 3ꞌ-UTR 23, 36, 37. Our bioinformatics analysis revealed the complementary binding sequence of miR-214-3p in 3′-UTR of DKK3, indicating that miR-214-3p may directly interact with DKK3 to degrade it. Significantly down-regulated DKK3 expression was identified in human osteosarcoma tissues and cells. Our functional gain and loss experiments in the osteosarcoma cells further revealed that by down-regulating the DKK3 expression, miR-214-3p promoted active β-catenin accumulation and then nuclear translocation, which further activates the translation of LEF1 in nuclear, causing Wnt/β-catenin signaling activation in osteosarcoma cells, thus to promote oncogenesis and metastasis of osteosarcoma. Therefore, inhibiting Wnt/β-catenin signaling activity with DKK3 via down-regulating miR-214-3p is a promising strategy to develop novel treatments for osteosarcoma patients.

Recently, natural, nonsynthetic medicines derived from natural sources have attracted a greater worldwide interest owing to the better tolerance, minimum side effects, and less likely to result in dependency than the synthetic drugs, which has also made considerable progress for the treatment of malignant tumors 38. In present work, influence of cantharidin on osteosarcoma progression was investigated *in vivo* and *in vitro*. Studies in MG-63, MNNG/HOS and U-2 OS osteosarcoma cells have revealed that cantharidin could induce osteosarcoma apoptosis and cell cycle arrest at the G2/M phase 39, 40. However, biological functions and molecular mechanisms of cantharidin in osteosarcoma, including the accurate cell cycle arresting phase (G2 or M); the effect on colony formation, migration and invasion abilities, and EMT characteristics; the involvement of miR-214-3p/DKK3/GSK-3β/β-catenin/LEF1 signaling pathway; the *in vivo* (using the orthotopic xenograft mouse model) anti-osteosarcoma efficacy need to be further investigated. Our data demonstrated that cantharidin effectively inhibited the viability, colony formation, migration and invasion abilities; while induced apoptosis and cell cycle arresting at M phase of U-2 OS and 143B cells. EMT plays critical role in cancer metastasis and is significantly related to cancer cell migration and invasion 41. As the major cytoskeletal component of mesenchymal cells, Vimentin has been used as a marker to identify the mesenchymal characteristics in cancer cells. Our findings disclosed that cantharidin could reverse EMT via down-regulation of vimentin expression.

It has been reported that cantharidin inhibits gastric cancer cell invasion and migration by suppressing PI3K/Akt signaling pathway via CCAT1; and inhibits growth of Triple-Negative Breast Cancer Cells by inhibiting autophagy. However, the molecular mechanism of cantharidin in anti-osteosarcoma need to be further investigated. Furthermore, to elucidate mechanism of cantharidin in inhibiting osteosarcoma carcinogenesis and progression, we investigated expressions of DKK3, p-GSK-3β and active β-catenin in the total proteins, as well as the total β-catenin and LEF1 in the nuclear proteins of the cantharidin-treated osteosarcoma cells with or without CHIR99021, miR-214-3p over-expression or miR-214-3p silence. DKK3 is a Wnt ligand activity antagonist and acts as an extracellular Wnt/β-catenin inhibitor. β-catenin is ubiquitylated and degraded by the proteasome when phosphorylated by the GSK-3β 42. Active β-Catenin Rabbit mAb is an antibody to precisely detect endogenous β-catenin protein with residues, including Ser33, Ser37 and Tr41, not being phosphorylated by GSK-3β, therefore, is active in canonical Wnt signaling pathway. Residues (Ser33/Ser37/Tr41) are phosphorylated β-catenin protein cannot be identified by the Active β-Catenin mAb. CHIR99021 is the specific inhibitor of GSK-3β. Our results showed that cantharidin up-regulated DKK3, decreased miR-214-3p, p-GSK-3β, active β-catenin, nuclear β-catenin and LEF1 expressions in osteosarcoma cells.

However, the above mentioned molecular changed and inhibited effects on osteosarcoma progression caused by cantharidin were partially reversed by either addition of CHIR99021 or miR-214-3p over-expression, indicating specific contribution of the miR-214-3p/DKK3/GSK-3β/β-catenin/LEF1 axis in cantharidin inhibited osteosarcoma progression.

It has been reported that intravenously administrated cantharidin shows an area under the curve of 204 ± 24 h·ng/mL and elimination half-life of 0.69 ± 0.03 h in beagle dogs, using a gas chromatography-mass spectrometry method 43. Clinical studies have shown that cantharidin combined with chemotherapy improves the quality of life in non-small cell lung cancer patients, while is not related to more toxicities 44; cantharidin injection combined with chemotherapy improves clinical benefit response and quality of life, and reduces side effects of chemotherapy^45^, indicating the importance of strengthening the education of cantharidin in physicians, ensure the safety and effective application of cantharides and reduce the occurrence of toxic reactions in patients.

## Conclusion

We revealed that, as an oncogenic gene, miR-214-3p targets 3ꞌ-UTR of the DKK3 to degrade it, which causes an accumulation of the active β-catenin in cytoplasm of osteosarcoma cells and leads to an increased β-catenin nuclear translocation to further activate LEF1 translation, as a result, Wnt/β-catenin signaling is activated to promote osteosarcoma progression.

Cantharidin inhibits osteosarcoma cell proliferation and metastasis via specific down-regulation of miR-214-3p to prevent DKK3 from being degraded. The up-regulated DKK3 inhibits GSK-3β phosphorylation and increases GSK-3β phosphorylated β-catenin. Degradation of phosphorylated β-catenin limits β-catenin nuclear translocation to inactivate LEF1 translation, resulting in an inhibited Wnt/β-catenin signaling activation. This suggests that cantharidin may be a prospective natural drug candidate for osteosarcoma treatment by targeting the miR-214-3p/DKK3/GSK-3β/β-catenin/LEF1 axis.

## Supplementary Material

Supplementary figures.

## Abbreviations

DKK: Dickkopf Homolog
TCF: T-cell factor
DOX: doxorubicin
PVDF: polyvinylidene fluoride
BCA: bicinchoninic acid assay
SDS-PAGE: polyacrylamide gel electrophoresis
HRP: horseradish peroxidase
RT-qPCR: real-time quantitative PCR
EDTA: ethylenediaminetetraacetic acid
LEF1: lymphoid enhancer factor 1
FBS: fetal bovine serum
EMT: epithelial-mesenchymal transition
UTR: untranslated region
DMSO: dimethyl sulfoxide, NS. not statistically significant
