# Supplementary material for: Cantharidin inhibits osteosarcoma proliferation and metastasis by directly targeting miR-214-3p/DKK3 axis to inactivate β-catenin nuclear translocation and LEF1 translation
Source: Int J Biol Sci. 2021 Jun 16;17(10):2504–22. doi: 10.7150/ijbs.51638 (PMC8315017; doi:10.7150/ijbs.51638)
Supplement: Supplementary file 1 — Supplementary figures. [file ijbsv17p2504s1.pdf]

Supplementary Figure legends

Supplementary Figure 1. Chemical structure of cantharidin.

Supplementary Figure 2. Nuclear magnetic resonance (NMR) spectrum of cantharidin.

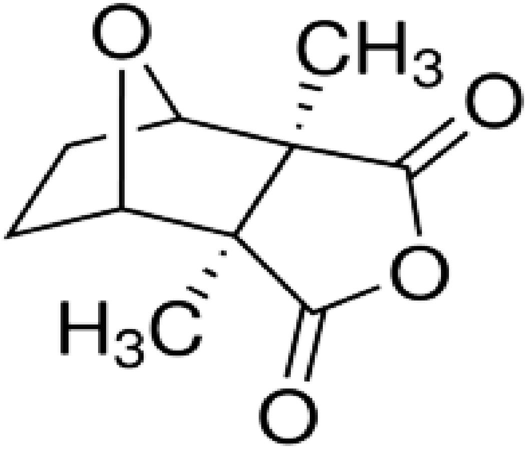

Supplementary Figure 1

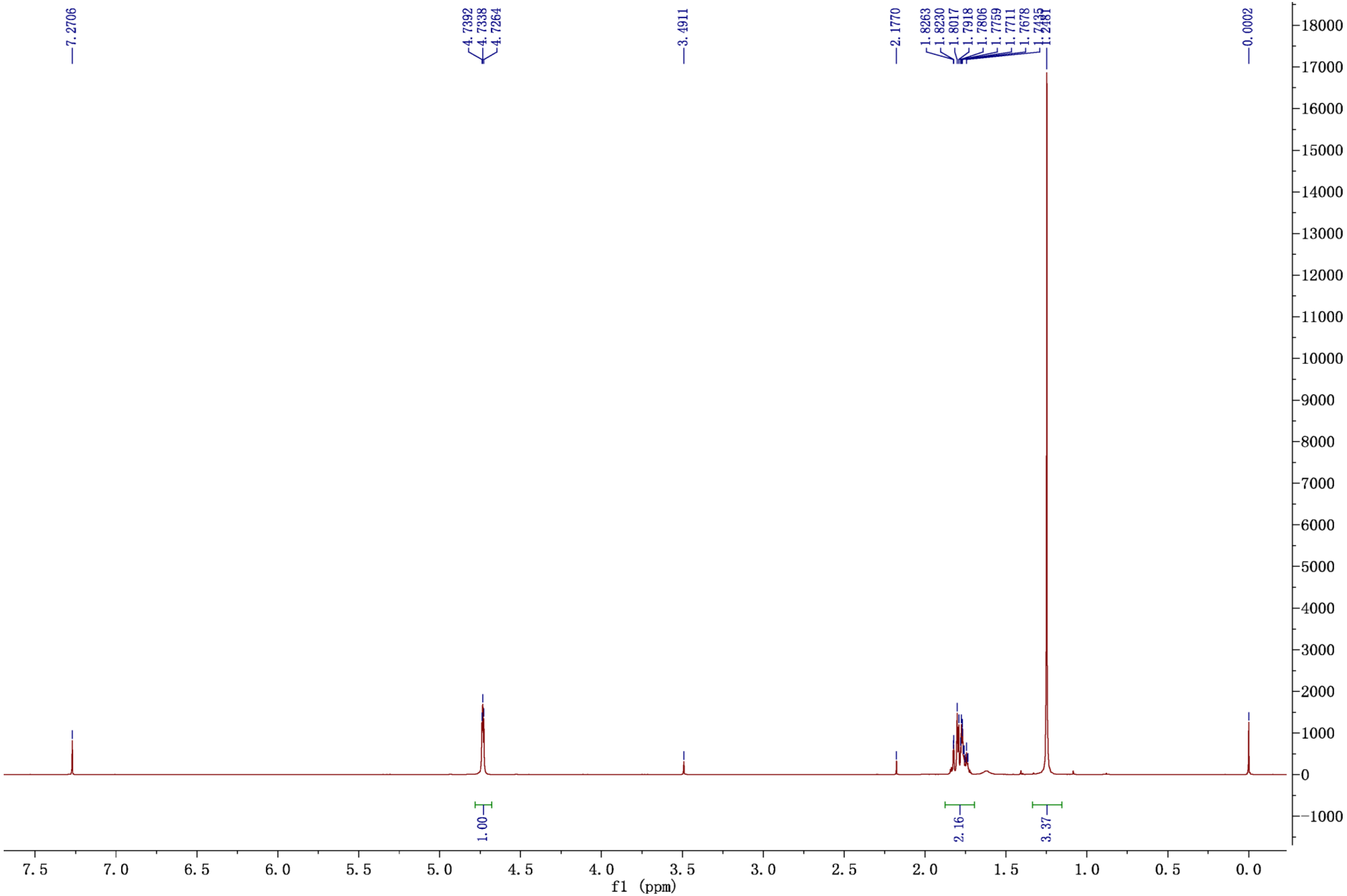

Supplementary Figure 2
